# Detection of Anti-ZIKV NS1 IgA, IgM, and Combined IgA/IgM and Identification of IL-4 and IL-10 as Potential Biomarkers for Early ZIKV and DENV Infections in Hyperendemic Regions, Thailand

**DOI:** 10.3390/tropicalmed8050284

**Published:** 2023-05-17

**Authors:** Vajee Petphong, Nathamon Kosoltanapiwat, Kriengsak Limkittikul, Pannamas Maneekan, Supawat Chatchen, Akanitt Jittmittraphap, Pimolpachr Sriburin, Siriporn Chattanadee, Pornsawan Leaungwutiwong

**Affiliations:** 1 Department of Microbiology and Immunology, Faculty of Tropical Medicine, Mahidol University, Bangkok 10400, Thailand; 2 Department of Tropical Pediatrics, Faculty of Tropical Medicine, Mahidol University, Bangkok 10400, Thailand; 3 Department of Tropical Hygiene, Faculty of Tropical Medicine, Mahidol University, Bangkok 10400, Thailand

**Keywords:** Zika virus, IgA, IgM, NS1 ELISA, cytokine, immunoassay

## Abstract

The frequency of Zika virus (ZIKV)-specific IgA and IgM and the cytokine expression profile of ZIKV-infected patients in hyperendemic areas remain unclear. This study investigated the rates of ZIKV non-structural protein 1 (NS1)-specific IgA and IgM and evaluated serum cytokine levels of ZIKV and Dengue virus (DENV) cases in Thailand to identify potential diagnostic biomarkers, elucidate the immunity against ZIKV and DENV, and investigate the association between cytokine levels and ZIKV symptoms. Low rates of positivity for ZIKV NS1-specific IgA and IgM were detected in our study. ZIKV NS1 IgA/M (11%, 11/101) in combination was more frequently detected than ZIKV NS1 IgM (2%, 2/101) or ZIKV NS1 IgA (4%, 4/96) alone, especially in acute ZIKV cases with previous DENV exposure (14%, 10/72). Cytokine analysis showed that both ZIKV and DENV infections induced polyfunctional immunity, and the latter triggered more prolonged responses. The existence of significant differences in IL-4 and IL-10 levels between acute ZIKV and acute DENV cases suggested that IL-4 (*p* = 0.0176) and IL-10 (*p* = 0.0003) may represent biomarkers for acute ZIKV and acute DENV infections, respectively. Analysis of the association between increased cytokine levels and ZIKV symptoms indicated that CXCL10 (*p* = 0.0029) was associated with exanthema, while IL-5 (*p* = 0.0496) was linked to headache. The detection of ZIKV NS1 IgA and IgM in combination may enhance the diagnosis of early ZIKV infection, particularly when levels of IgM or IgA alone are low or undetectable. IL-4 and IL-10 may serve as targets for the development of diagnostic tools to detect ZIKV and DENV infections early, respectively, in flavivirus-endemic regions.

## 1. Introduction

Zika virus (ZIKV) is a positive-sense, single-stranded RNA virus, belonging to the genus *Flavivirus*, family *Flaviviridae*. Dengue virus (DENV) is also a member of this family, and both ZIKV and DENV are found in tropical and subtropical regions where *Aedes* mosquitoes, their main vector, co-circulate [1]. Most ZIKV-infected patients are asymptomatic, whereas others exhibit mild symptoms resemble those of other arbovirus infections, especially DENV, making clinical-symptom-based diagnosis inadequate [2]. During major outbreaks in French Polynesia in 2013 and Brazil in 2015, ZIKV infection was linked to increased neurological complications, including Guillain–Barre syndrome in adults and microcephaly in infants [3]. Its transmission is expected to occur worldwide, especially through the mobility of infected mosquitoes and viremic travelers [4], thereby accentuating the demand for reliable and accurate diagnostic tools.

Despite the above background, current laboratory tests for diagnosing ZIKV have several limitations. Although reverse-transcription polymerase chain reaction (RT-PCR) assays, used for detecting ZIKV RNA during acute infection, represent the most specific tests for recent infections [5,6], their sensitivity is limited by a short viremic period and low-level viremia [7]. Serological tests, including IgM and IgG enzyme-linked immunosorbent assays (IgM and IgG ELISAs), are then used to assess antibody levels in patients with negative RT-PCR results; however, these assays’ specificity is affected by antibody cross-reactivity with other flaviviruses, making it challenging to interpret the results [8,9]. Thus, plaque reduction neutralization tests (PRNTs) are applied to acquire a confirmatory diagnosis of recent ZIKV infection in endemic areas via the detection of neutralizing antibodies; however, the assays may fail to confirm test results in populations previously infected with flavivirus as they cannot specify antibody classes and the presence of cross-reactive neutralizing antibodies [7]. Moreover, PRNTs are laborious to perform and can only be carried out at highly specialized laboratories, limiting their use in routine diagnostics [7]. Recently, several high-throughput and multiplex microsphere immunoassays, developed based on the use of flavivirus proteins, such as envelope protein and non-structural proteins NS1 and NS5, have been shown to detect multiple flavivirus infections in a single assay with accuracy comparable to that of ELISA [10,11]. The assay requires shorter testing time and lower sample volumes than ELISA; nevertheless, its utility and availability may be limited due to the requirements of skilled technicians, costly analysis instruments, and confirmation by PRNTs [10,11,12]. Studies have found that non-structural 1 (NS1)-based IgM and IgG ELISAs could detect flavivirus-specific antibodies with high sensitivity and specificity [13,14]. Interestingly, NS1-specific IgA has been shown to provide additional diagnostic value for acute ZIKV and DENV infections, even when the patient’s viremia has disappeared and IgM titers are low or absent, as found in secondary infections [15,16]. Morales et al. recently reported that anti-ZIKV NS1 IgA and IgM ELISA could boost the diagnosis of ZIKV infection in Brazil and Venezuela where arboviruses are endemic [17]. However, despite extensive studies on ZIKV antibodies, there have been few studies involving serological surveys of ZIKV-specific IgA and IgM in hyperendemic settings.

ZIKV exhibits multiple-cell tropism, infecting structural cells, including human skin cells [18] and immune cells, in the peripheral and central nervous systems [19]. Such infection triggers the release of immune mediators essential for viral clearance [20]. However, under chronic infection, the dysregulation of cytokine secretion can lead to excessive inflammation, causing cell death and tissue injuries, as seen in fetal microcephaly during pregnancy of ZIKV-infected women [21]. Recently, although CXCL10 has been suggested as a specific biomarker linked to neurological diseases in cases with confirmed ZIKV, the association between cytokine levels and Zika symptoms has not been consistently reported [22].

Interestingly, studies of cytokine profiles in patients with other flavivirus infections, including DENV and Japanese encephalitis virus (JEV), suggested that certain cytokines could serve as potential prognostic markers for disease severity and diagnostic markers of the disease [23,24,25,26,27,28]. Recent studies have suggested IFN-γ, IL-10, GM-CSF, and MIP-1β as potential predictor for severity of dengue disease and IL-10 as a diagnostic marker for dengue fever [23,24]. However, the application of the specified markers may be limited in other patient populations due to heterogeneity in patient cohorts and methodologies used in the current literature, contributing to inconsistent outcomes [29]. Accordingly, it is possible that cytokines may also serve as targets for diagnostic development [28]; nevertheless, the precise biomarkers for distinguishing ZIKV infection from infection with the most closely related flavivirus, DENV, in hyperendemic areas have not yet been identified.

In this study, we used Euroimmun ZIKV NS1-based IgM, IgA, and combined IgA/IgM (or IgA/M) ELISAs to detect IgA and IgM in the sera of patients in Thailand. We evaluated the levels of seven cytokines in sequential serum samples of ZIKV and DENV cases, using multiplex immunoassay to characterize immunity to ZIKV and DENV, to determine potential diagnostic biomarkers, and to investigate the association between cytokine levels and Zika symptoms.

## 2. Materials and Methods

### 2.1. Ethics Statement, Collection of Human Specimens, and Clinical Data

The protocol of this study was reviewed and approved by the ethics committee of the Faculty of Tropical Medicine, Mahidol University, Thailand (FTM-EC; approval number MUMT 2021-025-01). Human sera were obtained from two sites: the Department of Tropical Pediatrics and the Department of Microbiology and Immunology (collected in 2007–2009 and 2017–2019, respectively), Mahidol University, Thailand. The enrolled patients were ≥4 years of age, who came to the hospital with clinical symptoms of low-grade fever, arthralgia, myalgia, and rash/exanthema with a duration of up to 7 days. The samples collected at enrollment (≤7 days after symptom onset) and ≥15 days after disease onset were characterized as acute-phase samples and convalescent-phase samples, respectively. Pregnant women were excluded in the study. Demographic data and clinical information recorded at enrolment including age, sex, self-reported symptoms, and seven days of fever were provided by the Hospital for Tropical Diseases, Bangkok, Thailand. All sera were stored at −80 °C until used for all experiments in this study.

### 2.2. Sample Classification and Study Design

The serum samples were classified into three groups: ZIKV-infected samples, DENV-infected samples, and healthy samples, based on laboratory confirmation using real-time RT-PCR and ELISAs. The samples with positivity for ZIKV in real-time RT-PCR and/or IgM/IgG ELISAs, but negativity for DENV in RT-PCR and IgM/IgG ELISAs, were specified as ZIKV-infected samples. (Note that, within this sample group, sera with positivity in ELISA for DENV1-4 NS1 IgG were denoted as ZIKV sera with previous DENV exposure, while those with negativity in ELISA for DENV1-4 NS1 IgG were referred to as ZIKV sera without previous DENV exposure). These samples were used to evaluate the positive reactivity rate of anti-ZIKV NS1 IgA/M, IgM, and IgA ELISA kits (Euroimmun, Lübeck, Germany). The samples with positivity for DENV in real-time RT-PCR and/or IgM/IgG ELISAs, but negativity for ZIKV in RT-PCR and IgM/IgG ELISAs, were specified as DENV-infected samples (serving as a reference control for cross-reactivity), while the samples with negativity for both ZIKV and DENV in real-time RT-PCR and IgM/IgG ELISAs were specified as flavivirus-negative samples (serving as a negative control). These latter samples were also regarded as healthy samples, all of which were used to evaluate the negative reactivity rate of the ZIKV IgA/M, IgM, and IgA ELISAs. Samples tested using the ZIKV ELISAs are described in Table 1 and Table 2. Briefly, the positive reactivity rates of the Anti-Zika Virus NS1 IgA/M, IgM, and IgA ELISA kits (Euroimmun) were examined using 101 acute and 30 convalescent ZIKV sera, whereas the corresponding negative reactivity rates were evaluated using 5 acute and 10 convalescent DENV sera and 10 healthy sera. Subsequently, the remaining sera obtained from ZIKV and DENV cases (during acute and convalescent phases), as well as that obtained from healthy donors, were used for cytokine analysis.

### 2.3. RNA Extraction and Real-Time RT-PCR for ZIKV and DENV1-4 Diagnosis 

Viral RNA was extracted using the QIAamp Viral RNA Mini Kit (Qiagen, Hilden, Germany), in accordance with the manufacturer’s instructions. RNA was eluted in 60 μL of AVE buffer and stored at −80 °C without further thawing until use. Real-time RT-PCR was performed on a CFX96^TM^ real-time system C1000 thermal cycler (Bio-Rad, Tokyo, Japan). ZIKV detection was performed using the Altona Diagnostics RealStar ZIKV RT-PCR test kit (Altona Diagnostics GmbH, Hamburg, Germany), as described elsewhere [6]. Real-time RT-PCR conditions for ZIKV were as follows: 20 min at 55 °C; 2 min at 95 °C; and then 45 cycles for 15 s at 95 °C, 45 s at 55 °C, and 15 s at 72 °C. Detection of DENV1-4 was performed using a Dengue Real^TM^ Genotype kit (Sacace Biotechnologies, Como, Italy), as described previously [30]. Real-time RT-PCR conditions for DENV1-4 were as follows: 30 min at 50 °C; 15 min at 95 °C; 5 cycles for 10 s at 95 °C, 40 s at 56 °C, and 20 s at 72 °C; and then 40 cycles for 10 s at 95 °C, 40 s at 54 °C, and 20 s at 72 °C.

### 2.4. ZIKV and DENV Serological Diagnosis

Serum was used to detect the presence of IgM and IgG antibodies against ZIKV and DENV using anti-ZIKV and anti-DENV IgM and IgG ELISA kits (Euroimmun), in accordance with the manufacturer’s instructions. The assays were based on the identification of antibodies against recombinant non-structural protein 1 (NS1) of ZIKV coated on microplate wells. A calibrator containing ZIKV/DENV-specific IgM or IgG included in the respective kits was tested in each run to define the cut-off ratio of the test. Outcomes were assessed by calculating the ratio of the extinction of the control/patient sample over the extinction of the calibrator. Samples with ratios > 1.1 were considered positive, whereas those with values < 0.8 were considered negative. Intermediate values (>0.8 and <1.1) were regarded as indeterminate/borderline.

### 2.5. DENV1-4 NS1 IgG In-House ELISA

To evaluate ZIKV patients’ immune status, DENV1-4 NS1 IgG ELISA was performed using acute ZIKV sera. The assay was performed in accordance with previous studies [31]. Briefly, ELISA plates were filled with DENV-1, DENV-2, DENV-3, and DENV-4 NS1 proteins (2000 ng of combined DENV1-4 NS1 proteins per well; 500 ng per serotype per well) in 1× PBS, while blank wells filled with 1× PBS were also included in each plate (used to subtract non-specific binding). The plates were then incubated at 37 °C overnight, washed with 1× PBS containing 1% Tween 20 (PBS-T) six times and blocked with 5% skim milk in PBS-T for 1 h at 37 °C. The plates were then washed and filled with diluted (1:100) serum and controls, followed by incubation at 37 °C for 1 h and then washing. The plates were supplemented with diluted (1:1500) goat-anti-human IgG antibody conjugated to horseradish peroxidase (KPL Inc., Gaithersburg, MD, USA) and incubated at 37 °C for 1 h. The plates were then washed and filled with the substrate SureBlue^TM^ (KPL Inc.), followed by incubation at room temperature for 30 min. Finally, the reactions were stopped using 0.4 M sulfuric acid. The optical density (OD) was measured at 450 nm. An acute serum:negative control ELISA OD ratio of ≥2.0 was considered seropositive.

### 2.6. Anti-ZIKV NS1 IgA/M, IgM, and IgA ELISAs

To evaluate the positive and negative reactivity rates of serum samples, anti-ZIKV IgA/M, IgM, and IgA ELISAs (Euroimmun) were performed in accordance with the manufacturer’s instructions. These assays were based on the identification of antibodies against recombinant non-structural protein 1 (NS1) of ZIKV coated on microplate wells. A calibrator containing ZIKV-specific IgA/M, IgM, and IgA included in the corresponding kits was tested in each run to define the cut-off ratio of the test. Results were assessed by calculating the ratio of the extinction of the control/patient sample over the extinction of the calibrator. A sample was considered positive if its ratio was >1.1 and negative if it was <0.8. Intermediate values (>0.8 and <1.1) were interpreted as indeterminate/borderline.

### 2.7. Quantification of Cytokines Using Multiplex Immunoassay

The levels of seven biomarkers, namely IFN-γ, IL-4, IL-5, IL-6, IL-10, IL-21, and CXCL10, in the sera of ZIKV-infected patients, DENV-infected patients, and healthy controls were measured using a commercial kit (Human ProcartaPlex Mix and Match 7-plex Immunoassay; Thermo Fisher Scientific, Vienna, Austria) and a Luminex MAGPIX instrument (BIO-TECHNE, Minneapolis, MN, USA). Serum samples of 25 µL from all study subjects were used to perform the test, following the manufacturer’s instructions. The interpretation of cytokine concentrations was performed using ProcartaPlex Analyst 1.0 software (Thermo Fisher Scientific).

### 2.8. Statistical Analysis

All data were analyzed using GraphPad Prism 7 (Prism Software; GraphPad, San Diego, CA, USA). The significant differences between two groups of analytes were determined using Mann–Whitney test. Meanwhile, that of differences among the three groups of analytes were determined using the Kruskal–Wallis test. A *p*-value of less than 0.05 was considered statistically significant. Associations between cytokine levels and clinical symptoms were identified by Mann–Whitney U test (fold change of ≥2 and *p*-value < 0.05), to compare cytokine levels among healthy donors and ZIKV-infected patients with the absence or presence of specific symptoms. Associations between different immune mediators were determined by Spearman’s rank correlation test (*p*-value < 0.05 and an r_s_ range from −1 to +1). All significance correlations are presented with the degree of significance indicated (* *p* < 0.05, ** *p* < 0.01, *** *p* < 0.001, and **** *p* < 0.0001). All heatmaps were constructed using GraphPad Prism 7 (Prism Software; GraphPad, San Diego, CA, USA). In the heatmap display, the mean concentrations (pg/mL) for each measured cytokine in its corresponding group were computed, and the mean values were displayed for visualization. Additionally, the mean concentrations for each measured cytokine in its corresponding group were computed, then the mean values were subsequently scaled as percentages from 0 to 100% for visualization.

## 3. Results

### 3.1. Data of Participants and Samples

Among the 101 RT-PCR-confirmed ZIKV patients, there were 47 males and 54 females. The median age of all ZIKV patients was 28. The median number of days of fever of all ZIKV patients was 3. The analysis of DENV exposure among acute ZIKV cases showed that 29% (29/101) of the cases had never been exposed to dengue, whereas 71% (72/101) had. Notably, the observations of clinical manifestations available for 70 RT-PCR-confirmed ZIKV cases revealed that the most common symptom was exanthema (59/70, 84%), followed by fever (58/70, 83%), myalgia (23/70, 33%), headache (20/70, 29%), conjunctivitis (19/70, 27%), sore throat (18/70, 26%), arthralgia (14/70, 20%), and cough (14/70, 20%) (Table 1).

Regarding the numbers of RT-PCR-confirmed ZIKV serum samples used in this study, 71 sera were acute samples collected from 2017 to 2019, while 60 sera were paired samples of acute and convalescent phases (30 sera per phase), collected from 2007 to 2009. Upon confirmation using dengue and flavivirus diagnostic ELISA kits, 5 acute and 10 convalescent sera of DENV patients and 10 sera of healthy donors were used in this study (Table 2).

### 3.2. Detection of ZIKV IgA/M, IgM, and IgA in Confirmed ZIKV Cases in Thailand

Determination of Zika antibodies in all acute ZIKV (AZ) sera showed that 10% (10/101), 2% (2/101), and 3% (3/96) of them were positive for IgA/M, IgM, and IgA, respectively (Table 3). Notably, 14% (10/72), 3% (2/72), and 4% (3/69) of dengue-primed, acute sera were positive for IgA/M, IgM, and IgA, respectively, whereas no dengue-unprimed, acute sera were positive for any of the analyzed antibodies. However, considering all convalescent ZIKV (CZ) sera, IgA/M and IgA were detected at the same rate of 3% (1/30) of the samples, but IgM was undetectable (0%, 0/30). No dengue-primed, convalescent sera were positive for any of the analyzed antibodies, whereas 8% (1/13) of dengue-unprimed, convalescent sera were equivalently positive for IgA/M and IgA, while none of them were positive for IgM alone. Taking the findings together, the evaluation of Zika NS1-specific IgA/M, IgM, and IgA seroconversions in all ZIKV-infected patients revealed that 11% (11/101), 2% (2/101), and 4% (4/96) of them were positive for IgA/M, IgM, and IgA, respectively (Table 3). Specifically, the results show that 14% (10/72), 3% (2/72), and 4% (3/69) of dengue-primed, ZIKV patients were positive for IgA/M, IgM, and IgA, respectively. Conversely, 3% (1/29) and 4% (1/27) of dengue-unprimed ZIKV cases were positive for IgA/M and IgA, respectively, whereas none (0%, 0/29) of them were positive for IgM.

### 3.3. Comparison of Combined IgA/M, IgM, and IgA Reactivities against ZIKV NS1 ELISAs in Confirmed ZIKV Cases, Confirmed DENV Cases, and Healthy Donors

**Figure 1:**
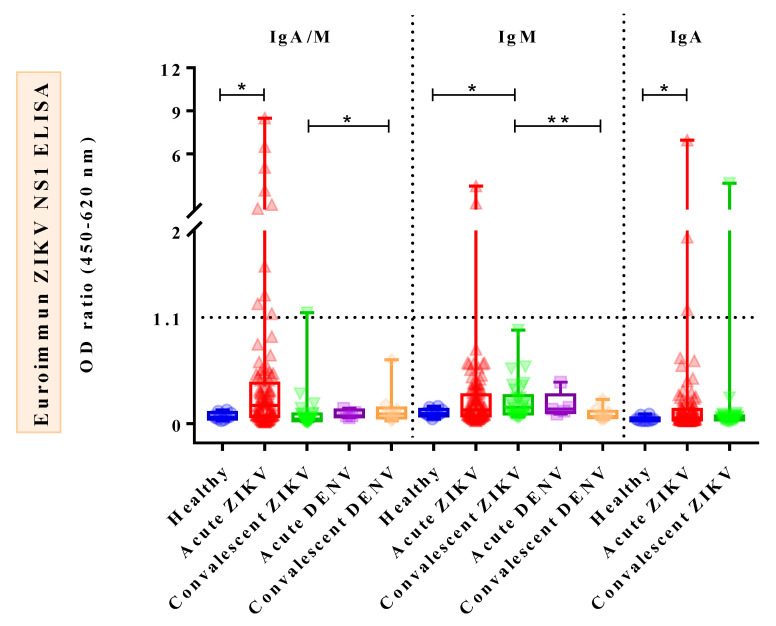
Anti–ZIKV NS1 IgA/IgM combined, IgM, and IgA reactivities in ZIKV cases compared to DENV cases and healthy donors. Boxplot demonstrates minimum, interquartile range, median, and maximum of each sample group on the *x*-axis. Note: The value of 1.1 on the *y*-axis represents cut–off value. Samples with cut–off values greater than 1.1 are considered positive. The asterisk (*) shows a comparison of the medians between two groups. All significant correlations are presented with the degree of significance indicated (* *p* < 0.05, ** *p* < 0.01).

**Figure 2:**
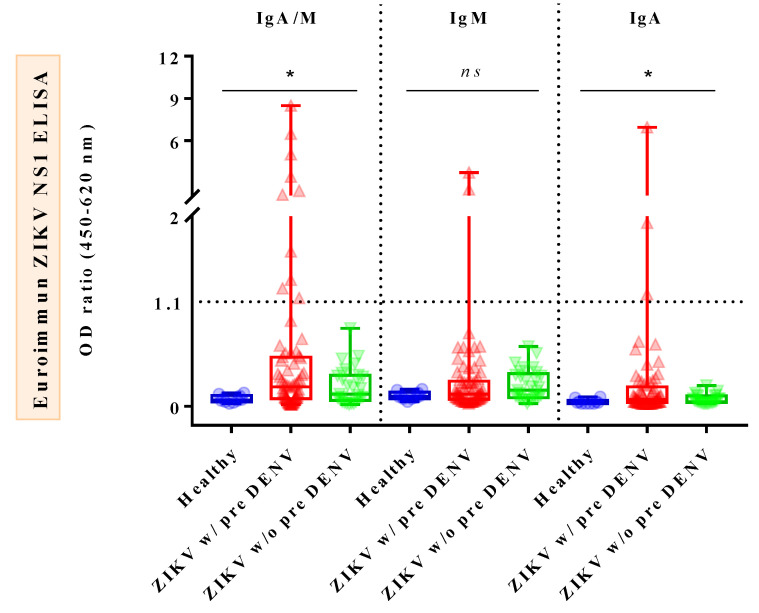
Differences in ZIKV–specific IgA/IgM combined, IgM, and IgA OD_450_ ratios among dengue–primed, ZIKV–infected patients, dengue–unprimed, ZIKV–infected cases, and healthy donors. Boxplot demonstrates minimum, interquartile range, median, and maximum of each sample group on the *x*-axis. Note: The value of 1.1 on the *y*-axis represents cut–off value. Samples with cut–off values greater than 1.1 are considered positive. The asterisk (*) or ns symbol shows a comparison of the medians of all three groups. ZIKV w/pre DENV represents ZIKV–infected cases with previous dengue infection, while ZIKV w/o pre DENV represents ZIKV–infected patients without previous dengue infection. All significant correlations are presented with the degree of significance indicated (* *p* < 0.05; ns, not significant).

Comparison of IgA/M reactivity against ZIKV IgA/IgM ELISA between different groups of subjects showed that IgA/M levels of AZ patients were significantly higher than those of healthy donors (HDs) (*p* = 0.0105), whereas those of CZ cases were significantly lower than those of convalescent DENV (CD) cases (*p* = 0.0252) (Figure 1, left). As for IgM reactivity against ZIKV IgM ELISA, the outcomes demonstrated that IgM levels of CZ cases were significantly higher than those of both CD cases (*p* = 0.0019) and HDs (*p* = 0.0251) (Figure 1, middle). Notably, there were no significant differences in IgA/M and IgM levels when AZ cases were compared with acute DENV (AD) cases. Regarding IgA reactivity against ZIKV IgA ELISA, IgA levels of AZ cases were significantly elevated when compared with HDs (*p* = 0.029), but not with CZ cases (*p* = 0.2245) (Figure 1, right). DENV sera were not subjected to this test due to a lack of samples. Interestingly, the comparison of IgA/M, IgM, and IgA OD_450_ ratios among the three subject groups, namely dengue-primed and dengue-unprimed ZIKV-infected patients and HDs, revealed that there were significant differences among the subjects for IgA/M (*p* = 0.024) and IgA (*p* = 0.037) OD_450_ ratios, but not for IgM OD_450_ ratios (*p* = 0.222) (Figure 2).

### 3.4. Cytokine Analysis of Zika and Dengue Patients in Endemic Country, Thailand

**Figure 3:**
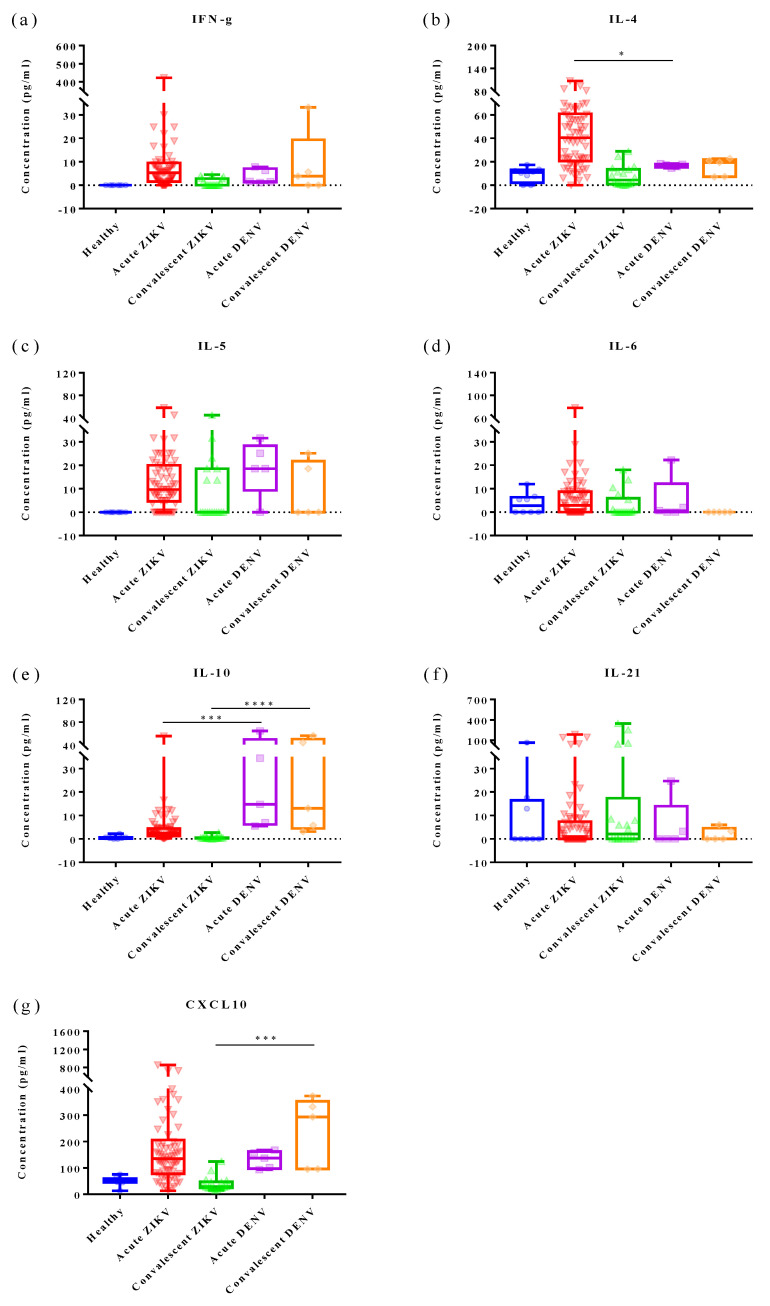
Differences in the levels of immune mediators in the acute and convalescent phases of ZIKV cases compared with DENV cases. Boxplot demonstrates minimum, interquartile range, median, and maximum of each sample group on the *x*-axis. The asterisk (*) shows a comparison of the medians between two groups. All significant correlations are presented with the degree of significance indicated (* *p* < 0.05, *** *p* < 0.001, **** *p* < 0.0001).

**Figure 4:**
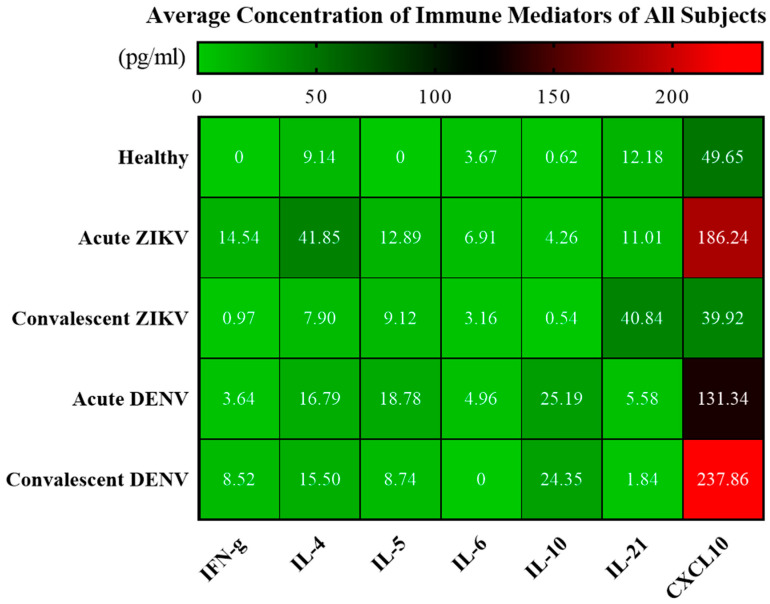
Heatmap showing average levels of cytokine/chemokine (pg/mL) in serum samples of ZIKV and DENV cases (in both acute and convalescent phases) compared to healthy donors.

**Figure 5:**
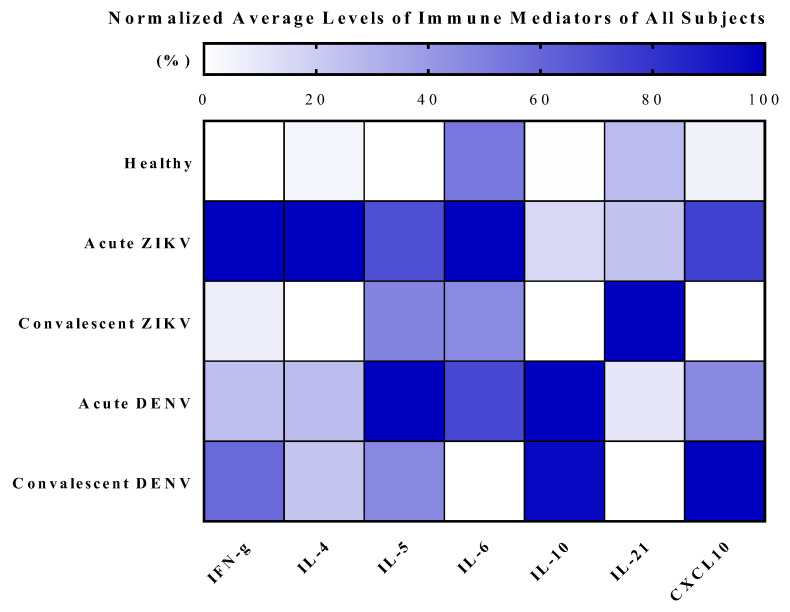
Heatmap showing relative expression of average levels of cytokine (percent; ranging from 0–100%) in serum samples of ZIKV and DENV cases (in both acute and convalescent phases) compared to healthy donors. Note: the darkest blue square indicates 100% (representing the highest concentration (pg/mL) of immune mediators in each column), while the white square indicates 0% (corresponding to the lowest concentration (pg/mL) of immune mediators in each column).

Among the seven cytokines analyzed, the results showed significant increases in IFN-γ (*p* < 0.0001), IL-4 (*p* < 0.0001), IL-5 (*p* < 0.0001), IL-10 (*p* < 0.0001), and CXCL10 (*p* = 0.0002) levels in AZ cases, compared with those in HDs (Figure 3, Figure 4 and Figure 5; Table 4 and Table 5; Supplementary Figure S1). Notably, CXCL10 exhibited the highest concentration levels in AZ sera, followed by IL-4, IFN-γ, IL-5, IL-21, IL-6, and IL-10, in that order; all declined to healthy levels in the CZ phase, with the exception of IL-21. Similarly, significant increases in IFN-γ (*p* = 0.0008), IL-10 (*p* = 0.0008), CXCL10 (*p* = 0.0016), IL-5 (*p* = 0.0070), and IL-4 (*p* = 0.0109) levels were observed in AD cases compared with those in HDs (Figure 3, Figure 4 and Figure 5; Table 4 and Table 5; Supplementary Figure S2). In contrast to the findings in CZ cases, IL-10 (*p* = 0.0008), CXCL10 (*p* = 0.0016), and IFN-γ (*p* = 0.0350) levels of CD cases were significantly higher than in HDs. Interestingly, IL-4 levels (*p* = 0.0176) of AZ patients were significantly higher than in AD cases, whereas IL-10 levels (*p* = 0.0003) of AZ cases were significantly lower than in AD cases (Figure 3). Notably, IL-10 (*p* < 0.0001) and CXCL10 (*p* = 0.0002) levels of CZ patients were significantly lower than those of CD cases (Figure 3).

### 3.5. Association between Cytokines and Symptoms in Acute ZIKV-Infected Patients

Investigation of the links between increased cytokine levels and certain symptoms presented by AZ patients, compared with individuals without symptoms, revealed associations between fever and cytokines, namely IFN-γ (*p* < 0.0001), IL-4 (*p* = 0.0106), IL-5 (*p* = 0.0018), and IL-10 (*p* = 0.0004). Moreover, headache was found to be associated with IL-5 (*p* = 0.0496). Interestingly, exanthema was strongly associated with IFN-γ (*p* = 0.0017), IL-4 (*p* = 0.0018), IL-5 (*p* = 0.0047), and CXCL10 (*p* = 0.0029). Meanwhile, conjunctivitis was linked to elevated levels of IFN-γ (*p* = 0.0306), IL-4 (*p* = 0.0112), and CXCL10 (*p* = 0.0332). Arthralgia was associated with high IL-4 levels (*p* = 0.0388), whereas myalgia was linked to increased IL-10 levels (*p* = 0.0114) (Table 6).

### 3.6. Correlations among Cytokine Levels in Acute ZIKV-Infected Patients

Spearman’s rank correlation coefficient (r_s_) analysis of elevated levels of the seven circulating cytokines during AZ infection revealed that IFN-γ was strongly correlated with IL-4 (r_s_ = 0.60, *p* < 0.001) and IL-10 (r_s_ = 0.53, *p* < 0.001), while being moderately correlated with IL-6 (r_s_ = 0.43, *p* < 0.001), CXCL10 (r_s_ = 0.42, *p* < 0.001), and IL-5 (r_s_ = 0.40, *p* < 0.001). In addition, CXCL10 was strongly correlated with IL-4 (r_s_ = 0.65, *p* < 0.001) and moderately linked to IL-21 (r_s_ = 0.42, *p* < 0.001). Moreover, IL-4 was moderately correlated with IL-6 (r_s_ = 0.47, *p* < 0.001) and IL-21 (r_s_ = 0.47, *p* < 0.001). Furthermore, weak positive correlations were found between CXCL10 and IL-6 (r_s_ = 0.37, *p* = 0.002); CXCL10 and IL-10 (r_s_ = 0.36, *p* = 0.002); IL-6 and IL-21 (r_s_ = 0.39, *p* < 0.001); IL-5 and IL-10 (r_s_ = 0.40, *p* < 0.001); and IL-5 and IL-21 (r_s_ = 0.27, *p* = 0.026) (Table 7).

## 4. Discussion

Zika virus (ZIKV) belongs to the genus *Flavivirus* (family *Flaviviridae*), which also includes DENV and JEV, all of which are endemic in Southeast Asia [1,3,25,32,33]. These viruses not only share a high degree of sequence and structural homology, but also cause similar clinical symptoms, making diagnosis of a specific virus infection difficult, especially in highly endemic regions where secondary infections are prevalent [1,3,25,32,33]. Although most ZIKV-infected patients develop mild illnesses, the virus infection can also lead to serious neurological diseases in certain cases, particularly in older adults and infants [2,3].

Thus, it is important to accurately identify the infecting virus and determine whether a patient contracted a primary or secondary flavivirus infection because knowing these details can help avoid unnecessary medical analysis and treatment and prevent adverse outcomes in vulnerable populations, especially in pregnant women who might give birth to a baby with neurological abnormalities and impaired growth if infected with ZIKV and in ZIKV-infected men who might spread the virus via sexual activities if unprotected [14,16,34,35]. Importantly, timely diagnosis can provide early warning of flavivirus epidemics to enhance management of patients and outbreaks. Owing to the limitations of current diagnostic tools [7,8,9], it is critical to find alternative methodologies for specific identification of a flavivirus infection.

The serological findings in this study suggest that the combined detection of both ZIKV NS1-specific IgA and IgM (IgA/M) could be more beneficial for early ZIKV diagnosis than the detection of either of these immunoglobulins alone. Using ZIKV NS1-based ELISAs, we found that IgA/M was more frequently detectable than either IgM or IgA alone in acute ZIKV sera, as indicated by the higher rate of positivity for IgA/M (14%, 10/72) than those of IgM (3%, 2/72) or IgA (4%, 3/69) among dengue-primed, acute ZIKV sera; nevertheless, none of the respective antibodies were detectable in all dengue-unprimed, acute ZIKV sera. However, the possibility of false-positive results due to flavivirus-induced antibody cross-reactivity could not be completely ruled out as the occurrence of cross-reactive antibodies against IgM assays among ZIKV cases with dengue (or other flaviviruses) background immunity has been extensively reported [9]. Notably, only 3% (1/30) of convalescent ZIKV sera were equally positive for IgA/M and IgA, whereas none (0%, 0/30) of them were positive for IgM. These results suggest that the acute serum may serve as a more suitable specimen for the Zika NS1-specific detection of IgA and IgM than the convalescent serum and that the combined detection of Zika NS1-specific IgA/M may enhance acute ZIKV identification, particularly in populations previously infected with dengue.

Nevertheless, it is worth noting that the low rates of positivity for Zika IgM were expected in this study as the acute sera were collected during viremia when it might be too early for IgM seroconversion to have occurred [9]. However, the absence of Zika IgM even in the convalescent samples might imply secondary/flavivirus-primed ZIKV infection in our cohort; correspondingly, 71% (72/101) of the cases had been exposed to DENV infection [16]. We hypothesized that the prevalence of anti-dengue IgG from previous infections might interfere with IgM or IgA seroconversion of current ZIKV infection, making the rates of positivity for IgM or IgA appear much lower than those of IgA/M combined. Similarly, recent studies showed that no or low levels of IgM were observed among early viremic (0% to 31.6%) and acute sera (0% to 48.4%) of ZIKV-confirmed cases in flavivirus-endemic countries, the convalescent sera of whom also showed a low rate of positivity for IgM of 12.5% [14,16,36]. Conversely, our results are in contrast to the observations among returned travelers with RT-PCR-confirmed primary ZIKV infection, in whom early and strong IgM responses (87.5% to 100%) were detected [14,36]. It has been suggested that the serological response to ZIKV may be altered by previous flavivirus infection [14,36]. Hence, the data presented here should be interpreted with caution.

Interestingly, through an analysis of antibody reactivity against the assays, we found significant differences in IgA/M (*p* = 0.0105) and IgA (*p* = 0.029) levels of acute ZIKV cases compared with those of HDs. We also observed significant differences in IgA/M (*p* = 0.024) and IgA (*p* = 0.037) levels, but not in IgM levels (*p* = 0.222), among acute ZIKV patients with and without previous dengue exposure and HDs. These results suggest that Zika NS1-specific IgA might be an alternative or additional diagnostic target to IgM for early ZIKV detection, particularly in dengue-primed individuals. Several reports are consistent with our findings regarding the better performance of IgA/M- and IgA-based tests over IgM-based ones [17]. Using acute samples, Morales et al. reported that an anti-ZIKV NS1 IgA/M test detected 15% of RT-PCR-confirmed ZIKV cases, whereas an anti-ZIKV NS1 IgM test detected only 9% of them. They also reported that, using follow-up samples, the IgA/M test (94%) outperformed the IgM (30%) and IgG (72%) tests; however, in that study, the history of previous flavivirus infection of participants was not explicitly clarified [17]. Using NS1-based ELISAs in a Latin American setting, Bozza et al. found that Zika IgA (53%) was more detectable than Zika IgM (33%), although the sample size was small [37]. Using ZIKV NS1-based IgA and IgM ELISAs to test paired serum samples from 31 Dominican patients with RT-PCR-confirmed ZIKV infection, Warnecke et al. reported that ZIKV NS1 IgA and IgM were detected in acute samples (collected 8–16 days after illness onset) with sensitivities of 93.5% and 48.4%, respectively, but were negative in all of viremic samples, collected 2–5 days after illness onset [16]. Their findings also showed that the proportion of positive IgA, but negative IgM cases, was higher in (flavivirus-primed) secondary (61.9%) than in primary (30.0%) ZIKV infections [16]. Likewise, a similar serological response has been observed among patients with DENV, in whom dengue NS1-specific IgA was more frequently detected in acute sera, and of greater prevalence during secondary DENV infection, compared with dengue NS1-specific IgM [38,39,40]. This information highlights the diagnostic value of IgA for the development of screening tests and detection tools to be used in endemic areas where primary and secondary flavivirus infections are prevalent and the value of IgM and IgG testing is lowered by low/undetectable IgM levels and high cross-reactivity against other flaviviruses [16,37]. Additionally, Zika IgA was reportedly detected in saliva during acute and early convalescent infection, making this specimen applicable for noninvasive diagnosis [41]. However, based on the findings mentioned above, the performance and efficiency of the IgA and IgA/IgM-based tests may be affected by the transient presence of IgA and IgM in blood circulation and variability in immune status among different patient populations [14,16,17].

In our study, we detected 100% negative rates in all of our control groups (dengue and healthy cases) using the Euroimmun ZIKV NS1-based ELISAs, corresponding to the high specificity of the tests (85% to 100%) observed in other studies [16,17]. Moreover, similar to the findings of Kikuti et al., we also found that the infecting serotype or prior dengue infection status (primary or secondary infection) did not have any effects on the specificity of the tests [36]. Recently, few studies have demonstrated the diagnostic potential of Zika NS1 IgA for acute ZIKV identification due to its complementary detectability to IgM [16,41]. Other studies have shown the benefits of the combined IgA and IgM test for acute ZIKV diagnosis in endemic areas where there is a high prevalence of flavivirus-primed individuals, in whom IgM titers are often transient and IgG levels are rapidly elevated [9,17]. According to the characterization of Zika IgA and IgM in sera collected from RT-PCR-confirmed ZIKV cases, living in a flavivirus-hyperendemic country in Southeast Asia, Thailand [42], we concluded that Zika NS1 IgA/M (11%, 11/101) was more frequently detected than Zika NS1 IgM (2%, 2/101) or Zika NS1 IgA (4%, 4/96) alone, particularly in acute ZIKV cases with previous DENV infection (14%, 10/72), as opposed to those without previous DENV exposure (3%, 1/29). We also concluded that the ZIKV NS1-based IgA/M ELISA may serve as an additional diagnostic tool for acute ZIKV detection, in addition to the use of RT-PCR and IgM ELISAs. As a limitation of the study, cross-reactivity tests against other flaviviruses were not performed. Further studies with larger numbers of well-characterized samples (including several types of samples such as serum, saliva, and urine) with a longer follow-up and cross-reactivity tests against other flaviviruses are warranted to properly address the kinetics of ZIKV NS1-specific IgA and IgM antibody responses in hyperendemic countries.

Regarding the investigation of the systemic immune activation profile of ZIKV and DENV cases in a hyperendemic setting (Thailand), our findings showed that both ZIKV and DENV infections induced polyfunctional systemic immune activation during the acute phase, as reflected by heightened cytokine levels associated with various T cell responses, including type-1 T helper or Th1 (IFN-γ, CXCL10), Th2 (IL-4, IL-5, IL-10), regulatory T or Treg (IL-10), as well as both proinflammatory (IL-5, IL-6, CXCL10) and immunoregulatory (IL-4, IL-10) responses, compared with those in healthy controls, coinciding with the findings in previous studies [43,44,45]. In fact, accumulating evidence has suggested that significant increases in IFN-γ and CXCL10 levels during acute ZIKV infection are desirable for adequate induction of antiviral response, resulting in a mild illness [43,46,47]. Notably, together with IL-23, via the activation of the Stat3 transcription factor, IL-6 is needed for the differentiation of Th17 cells that, when optimally activated, could help boost immune responses against invading viruses [48,49]. Accordingly, it has been reported that high levels of IFN-γ, IL-6, and IL-8 have been found in dengue-infected patients, in whom significant levels of IL-6 and IL-8 were linked to dengue hemorrhagic fever (DHF), and that the mean levels of these three cytokines were lower in primary dengue infection cases than in secondary ones [50,51,52].

In this study, during the convalescent phase of ZIKV infection, serum levels of most immune mediators generally returned to the normal state, consistent with the findings of Tappe et al., although the levels of several molecules (IL-6, IL-10, CXCL10) in that previous study remained significantly high in the recovery phase [53]. This discrepancy in cytokine levels may be attributable to the differences in ethnicity, as previously seen in DENV infection, because our cohort mostly consisted of Thai patients, while that in the study of Tappe et al. consisted of European travelers who had returned home [53,54]. Another possible explanation for this issue may be due to differences in immune status of individuals, which may be influenced by age and previous flavivirus infections [52,55,56], as the median age of ZIKV patients in our cohort (28 years) was lower than that of Tappe et al.’s study cohort (41 years) and most of the ZIKV-confirmed cases in our study were previously exposed to DENV infections, whereas those of Tappe et al.’s study suffered from primary ZIKV infection. Notably, the significant increases in IFN-γ, IL-4, IL-10, and CXCL10 levels during acute ZIKV infection could reflect the presence of the virus in blood circulation or other tissue reservoirs, as opposed to the levels that returned to the normal state in the convalescent phase when the viremia often becomes undetectable in plasma and urine [43,57]. In contrast, during convalescent DENV infection, the mean levels of IFN-γ, CXCL10, IL-4, and IL-10 remained relatively high, suggesting that the sustained interplay of Th1, Th2, and Treg may be needed for dengue control in this setting. Alternatively, it may reflect the migration of dengue-specific T cells, which reportedly move into the skin during acute infection and return to peripheral blood upon viral clearance [58,59]. However, owing to the small number of dengue cases in this study, larger cohorts of patients should be examined to strengthen the findings.

Notably, it is possible that pre-existing immunity to flavivirus could have influenced cytokine responses in our cohort as most of the subjects reside in a country where flavivirus is hyperendemic [55,56]. In fact, numerous studies have shown that, besides enhancing virus infection and worsening disease severity via a phenomenon known as “original antigenic sin,” both cross-reactive antibody and T cell responses caused by primary dengue infection could induce protective immunity upon secondary infection with different dengue serotypes and flaviviruses, including ZIKV [52,55,56]. Interestingly, more rapid and intense T cell responses against ZIKV have been reported in individuals with prior DENV infection, particularly cross-reactive T cell responses toward non-structural proteins, during primary infection [55,56].

Through analysis of potential biomarkers for differentially diagnosing ZIKV and DENV in endemic areas, our results suggest that IL-4 (*p* = 0.0176) could serve as a diagnostic target for distinguishing acute ZIKV from acute DENV infections, while IL-10 (*p* = 0.0003) could serve as a molecular marker for differentiating acute DENV from acute ZIKV infections, attributed to significant differences in the cytokine levels between the patient groups. Our additional experiments using a larger number of acute dengue cases helped support the results (Supplementary Figures S3 and S4; Supplementary Tables S1–S4). To our knowledge, this is the first study to observe the differences in cytokine levels in ZIKV and DENV cases in order to determine circulating molecules useful for the differential diagnosis of early ZIKV and DENV infections when a flavivirus infection is considered in hyperendemic areas. Evidently, IL-4 produced by Th2 cells and natural killer T (NKT) cells is critical for antiviral CD8^+^ T-cell (IFN-γ- and TNF-α–secreting cell) functions and antibody development during early viral infections, all of which are essential for inhibiting the establishment of persistent virus infections [60,61]. IL-4, functioning synergistically with IL-21, is required for normal germinal center function, B cell functions, and immunoglobulin (Ig) production and Ig class switching [61,62]. During early/acute viral infections, IL-10 produced by innate immune cells, including antigen-presenting cells (dendritic cells and macrophages) and NK cells, helps restrain excessive inflammation by counterbalancing proinflammatory signals triggered by virus infection, thereby reducing tissue damage [63]. However, in chronic virus infections (such as HCV, HBV, and HIV), elevated IL-10 levels during the acute phase have been linked to disease progression and persistent virus infections, mainly caused by T cell dysregulation [63]. Notably, previous studies showed that elevated IL-10 levels are associated with dengue pathogenesis and disease severity [52]. A study by Rathakrishnan et al. showed that dengue patients with warning signs (abdominal pain or tenderness, mucosal bleeding, clinical fluid accumulation, persistent vomiting, hepatomegaly, increase in hematocrit with rapid decrease in platelet count, and lethargy or restlessness) exhibited high levels of IL-10 throughout the disease course, whereas those without warning signs showed decreasing IL-10 levels toward a healthy state in the convalescent phase [44]. Moreover, Pérez et al. reported that increased IL-10 levels were detected in all subjects with DHF and that the patients with secondary infections showed consistently higher levels of IL-10 than the controls [64]. Conversely, Treg-produced IL-10 was reported to suppress Th17-induced inflammation in the tumor microenvironment in mice by preventing Th17 proliferation in the tumor and spleen [65]. Moreover, it is suggested that the combined functions of Treg cells, IL-10, and type I IFN were required for effective suppression of Th17-induced inflammation in the tumor microenvironment [65]. Thus, it is possible that the manipulation of Treg cells, by either suppressing or enhancing their suppressor functions, may represent a promising immunotherapy to prevent or alleviate severe dengue disease [52].

Considering the associations between cytokine levels and specific symptoms among acute ZIKV cases, we found that our patients presented with high frequencies of exanthema (84%), fever (83%), myalgia (33%), headache (29%), and conjunctivitis (27%). Interestingly, in agreement with the studies by Barros et al. and Sánchez-Arcila et al., reporting that exanthema in ZIKV-infected cases was associated with increased CXCL10 levels, our patients with exanthema showed significant elevations of CXCL10 (*p* = 0.0029), as well as IL-4 (*p* = 0.0018), IL-5 (*p* = 0.0047), and IFN-γ (*p* = 0.0017), in that order [43,45]. CXCL10 is a chemokine known to chemoattract CXCR3-positive cells, including activated T cells, NK cells, macrophages (microglia cells in CNS), and dendritic cells, toward sites of infection and inflammation [47]. Owing to levels of IL-4 and IL-5 being higher than IFN-γ levels, we hypothesize that the exanthema observed in our cohort might be attributable to the involvement of IgE-mediated hypersensitivity reactions driven by Th2-cytokine secretions (IL-4, IL-5, and IL-13), leading to an eosinophilic inflammatory reaction in the skin [66,67]. However, the IgE and eosinophil levels of the patients should be assessed to confirm these observations. Likewise, conjunctivitis found in our cohort was linked to significant increases in CXCL10 (*p* = 0.0332), followed by IL-4 (*p* = 0.0112) and IFN-γ (*p* = 0.0306) levels. Notably, these circulating molecules have been described to play critical roles in various allergic reactions (via cellular activation and lymphocyte/mast cell infiltration), including asthma and other types of conjunctivitis [47,68,69]. The fever in our cases was associated with significant escalations of IFN-γ (*p* < 0.0001), IL-10 (*p* = 0.0004), IL-5 (*p* = 0.0018), and IL-4 (*p* = 0.0106). IFN-γ has been suggested to regulate and mediate high fever in patients with blood cancers and infectious diseases [70,71]. Remarkably, headache in our study was associated with significantly high levels of IL-5 (*p* = 0.0496), corresponding to the findings of previous studies [43,45]. Moreover, arthralgia and myalgia in our patients were linked to increases in IL-4 (*p* = 0.0388) and IL-10 (*p* = 0.0114), respectively. The associations between the Zika symptoms and the increased levels of cytokines may suggest cytokines’ participation in the underlying mechanisms by which the symptoms are induced. In addition, the correlations in the levels of different cytokines observed in acute ZIKV cases may reflect the cytokines’ synergistic properties required for virus control and disease resolution.

## 5. Conclusions

In conclusion, the combined detection of ZIKV NS1-specific IgA and IgM appears to enhance the efficacy of acute ZIKV diagnosis, particularly in ZIKV cases with previous DENV exposure when the levels of either IgM or IgA alone are low or undetectable. In a flavivirus-hyperendemic country (Thailand), patients with acute ZIKV infection show polyfunctional systemic immune responses, which return to homeostasis in the convalescent phase, resulting in mild illness, whereas patients with acute DENV infection exhibit more prolonged polyfunctional immunity. The significant differences in cytokine levels detected among ZIKV and DENV cases suggest that IL-4 and IL-10 may serve as differential immune indicators for acute ZIKV and acute DENV infections, respectively, when a flavivirus infection is considered. The associations between specific symptoms and certain cytokines may suggest that cytokines participate in inducing Zika symptoms, making them applicable for symptom prediction and the development of treatment. Further studies, with a larger number of patients, well-characterized samples, and a longer follow-up, are necessary to properly address the kinetics of ZIKV-induced antibody responses and to identify biomarkers of a specific flavivirus infection. The findings in this study may be useful for the development of diagnostic tools required for more effective management of future outbreaks and treatments.

## Figures and Tables

**Table 1:** Characteristics of the ZIKV samples.

Characteristics of Samples	Microbiology and Immunology	Tropical Pediatrics	Total
Subject number (n)	71	30	101
Male:Female	0.82:1	1:1	0.87:1
Age (years old) *	36	9	27.41 ± 17.37 (28, 4–68)
Day of fever *	3	2	2.90 ± 1.49 (3, 1–7)
**Clinical symptoms** (n = 70):			
- Fever	58 (83%)	NA	58 (83%)
- Sore throat	18 (26%)	NA	18 (26%)
- Cough	14 (20%)	NA	14 (20%)
- Headache	20 (29%)	NA	20 (29%)
- Exanthema	59 (84%)	NA	59 (84%)
- Conjunctivitis	19 (27%)	NA	19 (27%)
- Arthralgia	14 (20%)	NA	14 (20%)
- Myalgia	23 (33%)	NA	23 (33%)
**Sample types:**			
Acute sample	71	30 **	101
Convalescent sample	0	30 **	30
Dengue exposure cases ***:			
Dengue IgG^+^	55 (77%)	17 (57%)	72 (71%)
Dengue IgG^−^	16 (23%)	13 (43%)	29 (29%)

* The values shown are mean ± SD (median, minimum–maximum). ** The data shown are paired blood samples from the Tropical Pediatrics lab. *** Acute serum samples were used to determine previous dengue exposure among ZIKV cases. NA, not available.

**Table 2:** Characteristics of DENV and healthy control samples.

Characteristics of Samples	Dengue Patients *		Healthy Donors (n = 10)
	Acute (n = 5)	Convalescent (n = 10)	
Dengue IgM^+^	5 (100%)	10 (100%)	NA
Dengue IgM^−^	0	0	NA
Dengue IgG^+^	0	10 (100%)	NA
Dengue IgG^−^	5 (100%)	0	NA
Flavivirus IgM^+^	NA	NA	0
Flavivirus IgM^−^	NA	NA	10 (100%)
Flavivirus IgG^+^	NA	NA	0
Flavivirus IgG^−^	NA	NA	10 (100%)

* The number of days of fever of all acute DENV patients was 1. NA = not available.

**Table 3:** Determination of rates of positivity and negativity for Zika IgA/IgM combined, IgM, and IgA by Euroimmun NS1-based ZIKV ELISAs.

Type of Samples/Cases	DENV IgG	Euroimmun ZIKV NS1 ELISAs		
		IgA^+^/IgM^+^	IgM^+^	IgA^+^
Acute ZIKV samples	Positive (n = 72)	10/72 (14%)	2/72 (3%)	3/69 (4%)
	Negative (n = 29)	0/29 (0%)	0/29 (0%)	0/27 (0%)
	Total (n = 101)	10/101 (10%)	2/101 (2%)	3/96 * (3%)
Convalescent ZIKV samples	Positive (n = 17)	0/17 (0%)	0/17 (0%)	0/17 (0%)
	Negative (n = 13)	1/13 (8%)	0/13 (0%)	1/13 (8%)
	Total (n = 30)	1/30 (3%)	0/30 (0%)	1/30 (3%)
ZIKV patients **	Positive (n = 72)	10/72 (14%)	2/72 (3%)	3/69 (4%)
	Negative (n = 29)	1/29 (3%)	0/29 (0%)	1/27 (4%)
	Total (n = 101)	11/101 (11%)	2/101 (2%)	4/96 * (4%)
Acute DENV samples	Total (n = 5)	0/5 (0%)	0/5 (0%)	NA
Convalescent DENV samples	Total (n = 10)	0/10 (0%)	0/10 (0%)	NA
Healthy samples	Total (n = 10)	0/10 (0%)	0/10 (0%)	0/10 (0%)

* Overall, 96 out of 101 acute ZIKV sera were tested by ZIKV NS1 IgA ELISA (5 went untested due to insufficient samples). ** The positive rates of the acute and convalescent ZIKV samples were not always derived from the same patients, so the positive rates in individual ZIKV patients were analyzed. NA, not available.

**Table 4:** Changes in the levels of circulating immune mediators in the acute and convalescent phases of infection among ZIKV-infected patients and DENV-infected patients compared with healthy donors.

Cytokine (pg/mL)	Type of Serum Samples				
	Acute ZIKV (n = 70)	Convalescent ZIKV (n = 18)	Acute DENV (n = 5)	Convalescent DENV (n = 5)	Healthy Donor (n = 8)
IFN-*γ*	14.54 ± 50.87/6.08 5.32 (0–422.70) 2.41, 26.67	1.13 ± 1.65/0.39 0 (0–4.49) 0.31, 1.95	3.64 ± 3.18/1.42 1.53 (1.10–7.82) −0.31, 7.59	8.52 ± 13.99/6.25 3.81 (0–33.15) −8.85, 25.88	0 ± 0/0 0 (0–0) 0, 0
IL-4	41.85 ± 24.85/2.97 40.44 (0–106.30) 35.93, 47.78	7.90 ± 8.77/2.07 4.41 (0–28.81) 3.54, 12.26	16.79 ± 1.54/0.69 17.28 (14.60–18.42) 14.87, 18.70	15.50 ± 7.66/3.43 19.43 (7.08–22.72) 5.99, 25.02	9.14 ± 6.22/2.20 10.88 (0–17.39) 3.94, 14.33
IL-5	12.90 ± 12.03/1.44 9.69 (0–58.09) 10.03, 15.76	9.12 ± 13.54/3.19 0 (0–44.85) 2.39, 15.85	18.78 ± 11.80/5.28 18.59 (0–31.60) 4.12, 33.43	8.74 ± 12.18/5.45 0 (0–25.10) −6.39, 23.87	0 ± 0/0 0 (0–0) 0, 0
IL-6	6.91 ± 12.02/1.44 2.89 (0–77.54) 4.05, 9.78	3.16 ± 5.60/1.32 0 (0–18.06) 0.38, 5.94	4.96 ± 9.68/4.33 0.60 (0–22.21) −7.06, 16.97	0 ± 0/0 0 (0–0) 0, 0	3.67 ± 4.42/1.56 2.70 (0–11.93) −0.03, 7.37
IL-10	4.26 ± 7.06/0.84 2.44 (0.24–55.16) 2.58, 5.95	0.54 ± 0.63/0.15 0.35 (0–2.64) 0.23, 0.85	25.20 ± 24.77/11.08 14.73 (5.47–64.38) −5.56, 55.95	24.35 ± 23.99/10.73 13.08 (3.13–56.09) −5.43, 54.14	0.63 ± 0.68/0.24 0.55 (0–2.18) 0.05, 1.20
IL-21	11.01 ± 32.27/3.86 0 (0–184.3) 3.32, 18.71	40.84 ± 97.16/22.90 2.07 (0–347.10) −7.48, 89.16	5.58 ± 10.76/4.81 0 (0–24.66) −7.78, 18.93	1.84 ± 2.69/1.20 0 (0–5.95) −1.51, 5.18	12.18 ± 23.18/8.20 0 (0–66.86) −7.20, 31.56
CXCL10	186.2 ± 170.2/20.35 135.2 (13.53–852.4) 145.60, 226.80	39.92 ± 27.69/6.53 29.13 (14.82–124.1) 26.15, 53.69	131.3 ± 33.16/14.83 137.2 (93.19–167.8) 90.17, 172.50	237.9 ± 132.7/59.34 293.0 (95.63–372.6) 73.11, 402.60	49.65 ± 17.52/6.19 51.05 (13.72–75.47) 35.00, 64.29

Data are the values (in pg per ml) of mean ± SD (standard deviation)/SEM (standard error of the mean), median (minimum–maximum), and 95% CI (confidence interval), respectively. The results were analyzed by Mann–Whitney U test.

**Table 5:** Differences in the levels of circulating immune mediators in the acute and convalescent phases of infection among ZIKV-infected patients, DENV-infected patients, and healthy donors.

Cytokine	*p*-Value ^a^ Derived from the Comparison between Each Group of Serum Samples					
	Acute ZIKV vs. Healthy Donor	Convalescent ZIVK vs. Healthy Donor	Acute DENV vs. Healthy Donor	Convalescent DENV vs. Healthy Donor	Acute ZIKV vs. Acute DENV	Convalescent ZIKV vs. Convalescent DENV
IFN-*γ*	<0.0001; ****	0.0721; *ns*	0.0008; ***	0.0350; *	0.3361; *ns*	0.1381; *ns*
IL-4	<0.0001; ****	0.6726; *ns*	0.0109; *	0.2688; *ns*	0.0176; *	0.0909; *ns*
IL-5	<0.0001; ****	0.0696; *ns*	0.0070; **	0.1282; *ns*	0.2132; *ns*	0.9026; *ns*
IL-6	0.5928; *ns*	0.6948; *ns*	>0.9999; *ns*	0.1608; *ns*	0.7391; *ns*	0.2006; *ns*
IL-10	<0.0001; ****	0.4187; *ns*	0.0008; ***	0.0008; ***	0.0003; ***	<0.0001; ****
IL-21	0.9495; *ns*	0.7600; *ns*	>0.9999; *ns*	0.6954; *ns*	0.8012; *ns*	0.3794; *ns*
CXCL10	0.0002; ***	0.1285; *ns*	0.0016; **	0.0016; **	0.9099; *ns*	0.0002; ***

^a^ Values of all significant correlations are given with the degree of significance indicated (* *p* < 0.05; ** *p* < 0.01; *** *p* < 0.001; **** *p* < 0.0001; *ns,* not significant); the results were analyzed by Mann–Whitney U test.

**Table 6:** Associations between the cytokine/chemokine levels and the absence or presence of specific clinical manifestations among acute ZIKV-infected patients (n = 60) and healthy donors (n = 8).

Cytokine (pg/mL) *p*-Value ^a^	Cytokine Levels among Individuals with the Absence or Presence of Clinical Symptoms							
	Fever	Sore Throat	Cough	Headache	Exanthema	Conjunctivitis	Arthralgia	Myalgia
	Absence (n = 18) vs. Presence (n = 50)	Absence (n = 55) vs. Presence (n = 13)	Absence (n = 56) vs. Presence (n = 12)	Absence (n = 49) vs. Presence (n = 19)	Absence (n = 19) vs. Presence (n = 49)	Absence (n = 52) vs. Presence (n = 16)	Absence (n = 55) vs. Presence (n = 13)	Absence (n = 49) vs. Presence (n = 19)
IFN-*γ*	0.77 (0–10.83) vs. 5.97 (0–422.70) <0.0001; ****	3.61 (0–422.7) vs. 6.42 (0–35.88) 0.2585; *ns*	3.81 (0–422.7) vs. 3.58 (0–48.29) 0.9081; *ns*	3.61 (0–56.64) vs. 7.83 (0–422.7) 0.1010; *ns*	0.67 (0–422.7) vs. 5.63 (0–50.66) 0.0017; **	3.41 (0–422.7) vs. 8.23 (0.9–29.94) 0.0306; *	3.41 (0–422.7) vs. 6.42 (0–29.94) 0.2455; *ns*	3.61 (0–422.7) vs. 6.12 (0–56.64) 0.1131; *ns*
IL-4	15.18 (0–69.94) vs. 45.1 (4–106.3) 0.0106; *	26.49 (0–106.3) vs. 55.78 (6.0–78.7) 0.0671; *ns*	39.29 (0–106.3) vs. 31.4 (3.9–85.1) 0.9079; *ns*	38.34 (0–106.3) vs. 38.82 (6.3–92.84) 0.6473; *ns*	17.43 (0–106.3) vs. 49.77 (6.0–92.84) 0.0018; **	23.6 (0–106.3) vs. 57.24 (11–92.84) 0.0112; *	29.94 (0–106.3) vs. 57.81 (3.89–92.84) 0.0388; *	28.58 (0–92.84) vs. 46.89 (4–106.3) 0.1381; *ns*
IL-5	0 (0–25.10) vs. 10.87 (0–58.09) 0.0018; **	8.53 (0–58.09) vs. 9.70 (0–31.60) 0.2189; *ns*	8.53 (0–58.09) vs. 10.28 (0–38.34) 0.9841; *ns*	8.53 (0.0–58.09) vs. 12.03 (0–38.34) 0.0496; *	0 (0–31.60) vs. 10.87 (0–58.09) 0.0047; **	8.53 (0–44.85) vs. 10.28 (0–58.09) 0.2223; *ns*	8.53 (0–58.09) vs. 12.02 (0–38.34) 0.3942; *ns*	8.53 (0–44.85) vs. 12.02 (0–58.09) 0.1299; *ns*
IL-6	2.52 (0–11.93) vs. 2.13 (0–77.54) 0.5793; *ns*	1.63 (0–77.54) vs. 5.04 (0–20.89) 0.5512; *ns*	2.13 (0–77.54) vs. 3.61 (0–20.89) 0.5558; *ns*	1.41 (0–37.86) vs. 3.62 (0–77.54) 0.4603; *ns*	5.40 (0–77.54) vs. 1.63 (0–28.84) 0.414; *ns*	0 (0–77.54) vs. 4.57 (0–28.84) 0.2589; *ns*	1.13 (0–77.54) vs. 5.04 (0–28.84) 0.5250; *ns*	1.63 (0–77.54) vs. 3.62 (0–37.86) 0.6509; *ns*
IL-10	1.35 (0–4.43) vs. 3.35 (0.2–55.2) 0.0004; ***	2.18 (0–55.16) vs. 3.92 (1.7–10.7) 0.0857; *ns*	2.44 (0–55.16) vs. 3.59 (0.3–12.1) 0.4476; *ns*	2.55 (0–55.16) vs. 2.50 (0.24–16.5) 0.3001; *ns*	2.28 (0–55.16) vs. 2.58 (0.24–16.50) 0.632; *ns*	2.995 (0–55.16) vs. 1.87 (0.90–12.14) 0.5214; *ns*	2.55 (0–55.16) vs. 1.61 (0.57–12.14) 0.5554; *ns*	2.18 (0–16.5) vs. 4.44 (1.3–55.2) 0.0114; *
IL-21	0 (0–66.86) vs. 0.70 (0–138.90) 0.5181; *ns*	0 (0–138.9) vs. 3.83 (0–18.51) 0.7609; *ns*	0 (0–66.86) vs. 2.05 (0–138.9) 0.6518; *ns*	2.32 (0–138.90) vs. 0 (0–10.58) 0.2113; *ns*	0 (0–138.9) vs. 0 (0–46.8) 0.9795; *ns*	0 (0–138.9) vs. 0 (0–46.80) 0.9228; *ns*	0 (0–138.9) vs. 4.10 (0–21.63) 0.1638; *ns*	0 (0–66.86) vs. 2.32 (0–138.9) 0.4379; *ns*
CXCL10	76.0 (14–525.6) vs. 140.9 (13.5–852) 0.0914; *ns*	120.4 (14–852.4) vs. 163.0 (46–727.6) 0.2267; *ns*	120.2 (14–852.4) vs. 169.3 (14–726.1) 0.4122; *ns*	120.6 (14–852.4) vs. 126.0 (23–399.2) 0.8922; *ns*	64.36 (13.5–852.4) vs. 151.2 (22.8–727.6) 0.0029; **	100.6 (13.5–852.4) vs. 156.2 (65.4–489.8) 0.0332; *	120.0 (13.7–852.4) vs. 143.2 (13.5–489.8) 0.5169; *ns*	115.7 (14–727.6) vs. 151.2 (14–852.4) 0.2649; *ns*

Note: Data are median (minimum–maximum), all presented in pg per ml, and respective *p*-value, respectively. ^a^ All significant correlations are presented with the degree of significance indicated (* *p* < 0.05, ** *p* < 0.01, *** *p* < 0.001, **** *p* < 0.0001, *ns* = not significant). The results were analyzed by Mann–Whitney U test.

**Table 7:** Correlations among cytokine levels in acute sera from RT-PCR-confirmed ZIKV cases.

Correlations among Immune Mediators in Acute ZIKV Subjects (n = 70)							
r_s_ 95% CI *p*-Value ^a^	IFN-γ	IL-4	IL-5	IL-6	IL-10	IL-21	CXCL10
IFN-γ	1						
IL-4	0.6038 0.42, 0.74 <0.001; ***	1					
IL-5	0.4017 0.18, 0.59 <0.001; ***	0.1923 −0.05, 0.41 0.111; *ns*	1				
IL-6	0.4282 0.21, 0.61 <0.001; ***	0.4676 0.25, 0.64 <0.001; ***	0.2123 −0.03, 0.43 0.078; *ns*	1			
IL-10	0.5266 0.33, 0.68 <0.001; ***	0.2271 −0.02, 0.44 0.059; *ns*	0.3990 0.17, 0.58 <0.001; ***	0.1961 −0.05, 0.42 0.104; *ns*	1		
IL-21	0.2019 −0.04, 0.42 0.094; *ns*	0.4730 0.26, 0.64 <0.001; ***	0.2663 0.03, 0.48 0.026; *	0.3873 0.16, 0.58 <0.001; ***	0.1770 −0.07, 0.40 0.143; *ns*	1	
CXCL10	0.4175 0.20, 0.60 <0.001; ***	0.6452 0.48, 0.77 <0.001; ***	0.1417 −0.10, 0.37 0.242; *ns*	0.3716 0.14, 0.56 0.002; **	0.3603 0.13, 0.55 0.002; **	0.4182 0.20, 0.60 <0.001; ***	1

Abbreviation: r_s_ = Spearman’s Rho (rs) or Spearman rank correlation measures the strength and direction of the relationship between two variables. ^a^ All significant correlations are presented with the degree of significance indicated (* *p* < 0.05, ** *p* < 0.01, *** *p* < 0.001, *ns* = not significant). The results were analyzed by Spearman’s rank correlation test.

## Data Availability

Data are reported in the current study and are available from the corresponding author upon request.

## Supplementary Materials

The following supporting information can be downloaded at: https://www.mdpi.com/article/10.3390/tropicalmed8050284/s1, Figure S1: Levels of immune mediators in the acute and convalescent phases of ZIKV infection in comparison to healthy donors; Figure S2: Levels of immune mediators in the acute and convalescent phase of DENV infection in comparison to healthy donors; Figure S3: Levels of IL-4 and IL-10 in acute ZIKV cases (n = 70) and acute DENV cases (n = 40) in comparison to healthy donors (n = 13); Figure S4: Differences in the levels of IL-4 and IL-10 in the acute and convalescent phases of ZIKV cases (acute, n = 70; convalescent, n = 18) compared with DENV cases (acute, n = 40; convalescent, n = 5); Table S1: Changes in levels of IL-4 and IL-10 in the acute phase of infection among ZIKV-infected and DENV-infected cases compared to healthy donors; Table S2: Differences in the levels of IL-4 and IL-10 in the acute phase of infection among ZIKV-infected patients, DENV-infected patients, and healthy donors; Table S3: Changes in levels of IL-4 and IL-10 in the acute and convalescent phases of infection among ZIKV-infected and DENV-infected cases compared to healthy donors; Table S4: Differences in the levels of IL-4 and IL-10 in the acute and convalescent phases of infection among ZIKV-infected patients and DENV-infected patients.

## References

[1] Heinz F.X., Stiasny K. (2017). The Antigenic Structure of Zika Virus and Its Relation to Other Flaviviruses: Implications for Infection and Immunoprophylaxis. Microbiol. Mol. Biol. Rev..

[2] Shuaib W., Stanazai H., Abazid A.G., Mattar A.A. (2016). Re-Emergence of Zika Virus: A Review on Pathogenesis, Clinical Manifestations, Diagnosis, Treatment, and Prevention. Am. J. Med..

[3] Relich R.F., Loeffelholz M. (2017). Zika Virus. Clin. Lab. Med..

[4] Hills S.L., Fischer M., Petersen L.R. (2017). Epidemiology of Zika Virus Infection. J. Infect. Dis..

[5] Judice C.C., Tan J.J.L., Parise P.L., Kam Y.W., Milanez G.P., Leite J.A., Caserta L.C., Arns C.W., Resende M.R., Angerami R. (2018). Efficient detection of Zika virus RNA in patients’ blood from the 2016 outbreak in Campinas, Brazil. Sci. Rep..

[6] L’Huillier A.G., Lombos E., Tang E., Perusini S., Eshaghi A., Nagra S., Frantz C., Olsha R., Kristjanson E., Dimitrova K. (2017). Evaluation of Altona Diagnostics RealStar Zika Virus Reverse Transcription-PCR Test Kit for Zika Virus PCR Testing. J. Clin. Microbiol..

[7] Munoz-Jordan J.L. (2017). Diagnosis of Zika Virus Infections: Challenges and Opportunities. J. Infect. Dis..

[8] Cabral-Castro M.J., Cavalcanti M.G., Peralta R.H.S., Peralta J.M. (2016). Molecular and serological techniques to detect co-circulation of DENV, ZIKV and CHIKV in suspected dengue-like syndrome patients. J. Clin. Virol. Off. Publ. Pan Am. Soc. Clin. Virol..

[9] Lanciotti R.S., Kosoy O.L., Laven J.J., Velez J.O., Lambert A.J., Johnson A.J., Stanfield S.M., Duffy M.R. (2008). Genetic and serologic properties of Zika virus associated with an epidemic, Yap State, Micronesia, 2007. Emerg. Infect. Dis..

[10] Tyson J., Tsai W.Y., Tsai J.J., Massgard L., Stramer S.L., Lehrer A.T., Nerurkar V.R., Wang W.K. (2019). A high-throughput and multiplex microsphere immunoassay based on non-structural protein 1 can discriminate three flavivirus infections. PLoS Negl. Trop. Dis..

[11] Wong S.J., Furuya A., Zou J., Xie X., Dupuis A.P., Kramer L.D., Shi P.Y. (2017). A Multiplex Microsphere Immunoassay for Zika Virus Diagnosis. EBioMedicine.

[12] Khalifian S., Raimondi G., Brandacher G. (2015). The use of luminex assays to measure cytokines. J. Investig. Dermatol..

[13] Tyson J., Tsai W.Y., Tsai J.J., Brites C., Mässgård L., Ha Youn H., Pedroso C., Drexler J.F., Stramer S.L., Balmaseda A. (2019). Combination of Nonstructural Protein 1-Based Enzyme-Linked Immunosorbent Assays Can Detect and Distinguish Various Dengue Virus and Zika Virus Infections. J. Clin. Microbiol..

[14] Steinhagen K., Probst C., Radzimski C., Schmidt-Chanasit J., Emmerich P., van Esbroeck M., Schinkel J., Grobusch M.P., Goorhuis A., Warnecke J.M. (2016). Serodiagnosis of Zika virus (ZIKV) infections by a novel NS1-based ELISA devoid of cross-reactivity with dengue virus antibodies: A multicohort study of assay performance, 2015 to 2016. Euro Surveill..

[15] Balmaseda A., Guzmán M.G., Hammond S., Robleto G., Flores C., Téllez Y., Videa E., Saborio S., Pérez L., Sandoval E. (2003). Diagnosis of dengue virus infection by detection of specific immunoglobulin M (IgM) and IgA antibodies in serum and saliva. Clin. Vaccine Immunol..

[16] Warnecke J.M., Lattwein E., Saschenbrecker S., Stöcker W., Schlumberger W., Steinhagen K. (2019). Added value of IgA antibodies against Zika virus non-structural protein 1 in the diagnosis of acute Zika virus infections. J. Virol. Methods.

[17] Morales I., Rosenberger K.D., Magalhaes T., Morais C.N.L., Braga C., Marques E.T.A., Calvet G.A., Damasceno L., Brasil P., Bispo de Filippis A.M. (2021). Diagnostic performance of anti-Zika virus IgM, IgAM and IgG ELISAs during co-circulation of Zika, dengue, and chikungunya viruses in Brazil and Venezuela. PLoS Negl. Trop. Dis..

[18] Hamel R., Dejarnac O., Wichit S., Ekchariyawat P., Neyret A., Luplertlop N., Perera-Lecoin M., Surasombatpattana P., Talignani L., Thomas F. (2015). Biology of Zika Virus Infection in Human Skin Cells. J. Virol..

[19] Mlakar J., Korva M., Tul N., Popovic M., Poljsak-Prijatelj M., Mraz J., Kolenc M., Resman Rus K., Vesnaver Vipotnik T., Fabjan Vodusek V. (2016). Zika Virus Associated with Microcephaly. N. Engl. J. Med..

[20] Medin C.L., Rothman A.L. (2017). Zika Virus: The Agent and Its Biology, With Relevance to Pathology. Arch. Pathol. Lab. Med..

[21] Noronha L., Zanluca C., Azevedo M.L., Luz K.G., Santos C.N. (2016). Zika virus damages the human placental barrier and presents marked fetal neurotropism. Mem. Inst. Oswaldo Cruz.

[22] Kam Y.W., Leite J.A., Lum F.M., Tan J.J.L., Lee B., Judice C.C., Teixeira D.A.T., Andreata-Santos R., Vinolo M.A., Angerami R. (2017). Specific Biomarkers Associated With Neurological Complications and Congenital Central Nervous System Abnormalities From Zika Virus-Infected Patients in Brazil. J. Infect. Dis..

[23] Patro A.R.K., Mohanty S., Prusty B.K., Singh D.K., Gaikwad S., Saswat T., Chattopadhyay S., Das B.K., Tripathy R., Ravindran B. (2019). Cytokine Signature Associated with Disease Severity in Dengue. Viruses.

[24] Puc I., Ho T.C., Yen K.L., Vats A., Tsai J.J., Chen P.L., Chien Y.W., Lo Y.C., Perng G.C. (2021). Cytokine Signature of Dengue Patients at Different Severity of the Disease. Int. J. Mol. Sci..

[25] Winter P.M., Dung N.M., Loan H.T., Kneen R., Wills B., Thu le T., House D., White N.J., Farrar J.J., Hart C.A. (2004). Proinflammatory cytokines and chemokines in humans with Japanese encephalitis. J. Infect. Dis..

[26] Kalita J., Srivastava R., Mishra M.K., Basu A., Misra U.K. (2010). Cytokines and chemokines in viral encephalitis: A clinicoradiological correlation. Neurosci. Lett..

[27] Abhishek K.S., Chakravarti A., Baveja C.P., Kumar N., Siddiqui O., Kumar S. (2017). Association of interleukin-2, -4 and -10 with dengue severity. Indian J. Pathol. Microbiol..

[28] Gowri Sankar S., Alwin Prem Anand A. (2021). Cytokine IP-10 and GM-CSF are prognostic biomarkers for severity in secondary dengue infection. Hum. Immunol..

[29] Lee Y.H., Leong W.Y., Wilder-Smith A. (2016). Markers of dengue severity: A systematic review of cytokines and chemokines. J. Gen. Virol..

[30] Tsai J.J., Liu W.L., Lin P.C., Huang B.Y., Tsai C.Y., Chou P.H., Lee F.C., Ping C.F., Lee P.A., Liu L.T. (2019). An RT-PCR panel for rapid serotyping of dengue virus serotypes 1 to 4 in human serum and mosquito on a field-deployable PCR system. PLoS ONE.

[31] Sriburin P., Sittikul P., Kosoltanapiwat N., Sirinam S., Arunsodsai W., Sirivichayakul C., Limkittikul K., Chatchen S. (2021). Incidence of Zika Virus Infection from a Dengue Epidemiological Study of Children in Ratchaburi Province, Thailand. Viruses.

[32] Al-Qahtani A.A., Nazir N., Al-Anazi M.R., Rubino S., Al-Ahdal M.N. (2016). Zika virus: A new pandemic threat. J. Infect. Dev. Ctries..

[33] Priyamvada L., Quicke K.M., Hudson W.H., Onlamoon N., Sewatanon J., Edupuganti S., Pattanapanyasat K., Chokephaibulkit K., Mulligan M.J., Wilson P.C. (2016). Human antibody responses after dengue virus infection are highly cross-reactive to Zika virus. Proc. Natl. Acad. Sci. USA.

[34] Saiz J.C., Vazquez-Calvo A., Blazquez A.B., Merino-Ramos T., Escribano-Romero E., Martin-Acebes M.A. (2016). Zika Virus: The Latest Newcomer. Front. Microbiol..

[35] Hamer D.H., Wilson M.E., Jean J., Chen L.H. (2017). Epidemiology, Prevention, and Potential Future Treatments of Sexually Transmitted Zika Virus Infection. Curr. Infect. Dis. Rep..

[36] Kikuti M., Tauro L.B., Moreira P.S.S., Campos G.S., Paploski I.A.D., Weaver S.C., Reis M.G., Kitron U., Ribeiro G.S. (2018). Diagnostic performance of commercial IgM and IgG enzyme-linked immunoassays (ELISAs) for diagnosis of Zika virus infection. Virol. J..

[37] Bozza F.A., Moreira-Soto A., Rockstroh A., Fischer C., Nascimento A.D., Calheiros A.S., Drosten C., Bozza P.T., Souza T.M.L., Ulbert S. (2019). Differential Shedding and Antibody Kinetics of Zika and Chikungunya Viruses, Brazil. Emerg. Infect. Dis..

[38] Shu P.Y., Chen L.K., Chang S.F., Yueh Y.Y., Chow L., Chien L.J., Chin C., Lin T.H., Huang J.H. (2000). Dengue NS1-specific antibody responses: Isotype distribution and serotyping in patients with Dengue fever and Dengue hemorrhagic fever. J. Med. Virol..

[39] Vázquez S., Pérez A.B., Ruiz D., Rodríguez R., Pupo M., Calzada N., González L., González D., Castro O., Serrano T. (2005). Serological markers during dengue 3 primary and secondary infections. J. Clin. Virol. Off. Publ. Pan Am. Soc. Clin. Virol..

[40] Groen J., Velzing J., Copra C., Balentien E., Deubel V., Vorndam V., Osterhaus A.D. (1999). Diagnostic value of dengue virus-specific IgA and IgM serum antibody detection. Microbes Infect..

[41] Zhao L.Z., Hong W.X., Wang J., Yu L., Hu F.Y., Qiu S., Yin C.B., Tang X.P., Zhang L.Q., Jin X. (2019). Kinetics of antigen-specific IgM/IgG/IgA antibody responses during Zika virus natural infection in two patients. J. Med. Virol..

[42] Yamanaka A., Matsuda M., Okabayashi T., Pitaksajjakul P., Ramasoota P., Saito K., Fukasawa M., Hanada K., Matsuura T., Muramatsu M. (2021). Seroprevalence of Flavivirus Neutralizing Antibodies in Thailand by High-Throughput Neutralization Assay: Endemic Circulation of Zika Virus before 2012. mSphere.

[43] Barros J.B.S., da Silva P.A.N., Koga R.C.R., Gonzalez-Dias P., Carmo Filho J.R., Nagib P.R.A., Coelho V., Nakaya H.I., Fonseca S.G., Pfrimer I.A.H. (2018). Acute Zika Virus Infection in an Endemic Area Shows Modest Proinflammatory Systemic Immunoactivation and Cytokine-Symptom Associations. Front. Immunol..

[44] Rathakrishnan A., Wang S.M., Hu Y., Khan A.M., Ponnampalavanar S., Lum L.C., Manikam R., Sekaran S.D. (2012). Cytokine expression profile of dengue patients at different phases of illness. PLoS ONE.

[45] Sánchez-Arcila J.C., Badolato-Correa J., de Souza T.M.A., Paiva I.A., Barbosa L.S., Nunes P.C.G., Lima M., Dos Santos F.B., Damasco P.V., da Cunha R.V. (2020). Clinical, Virological, and Immunological Profiles of DENV, ZIKV, and/or CHIKV-Infected Brazilian Patients. Intervirology.

[46] Le Page C., Génin P., Baines M.G., Hiscott J. (2000). Interferon activation and innate immunity. Rev. Immunogenet..

[47] Liu M., Guo S., Hibbert J.M., Jain V., Singh N., Wilson N.O., Stiles J.K. (2011). CXCL10/IP-10 in infectious diseases pathogenesis and potential therapeutic implications. Cytokine Growth Factor Rev..

[48] Wei L., Laurence A., Elias K.M., O’Shea J.J. (2007). IL-21 is produced by Th17 cells and drives IL-17 production in a STAT3-dependent manner. J. Biol. Chem..

[49] Wan Z., Zhou Z., Liu Y., Lai Y., Luo Y., Peng X., Zou W. (2020). Regulatory T cells and T helper 17 cells in viral infection. Scand. J. Immunol..

[50] Priyadarshini D., Gadia R.R., Tripathy A., Gurukumar K.R., Bhagat A., Patwardhan S., Mokashi N., Vaidya D., Shah P.S., Cecilia D. (2010). Clinical findings and pro-inflammatory cytokines in dengue patients in Western India: A facility-based study. PLoS ONE.

[51] Pandey N., Jain A., Garg R.K., Kumar R., Agrawal O.P., Lakshmana Rao P.V. (2015). Serum levels of IL-8, IFNγ, IL-10, and TGF β and their gene expression levels in severe and non-severe cases of dengue virus infection. Arch. Virol..

[52] Bhatt P., Sabeena S.P., Varma M., Arunkumar G. (2021). Current Understanding of the Pathogenesis of Dengue Virus Infection. Curr. Microbiol..

[53] Tappe D., Pérez-Girón J.V., Zammarchi L., Rissland J., Ferreira D.F., Jaenisch T., Gómez-Medina S., Günther S., Bartoloni A., Muñoz-Fontela C. (2016). Cytokine kinetics of Zika virus-infected patients from acute to reconvalescent phase. Med. Microbiol. Immunol..

[54] Restrepo B.N., Ramirez R.E., Arboleda M., Alvarez G., Ospina M., Diaz F.J. (2008). Serum levels of cytokines in two ethnic groups with dengue virus infection. Am. J. Trop. Med. Hyg..

[55] Subramaniam K.S., Lant S., Goodwin L., Grifoni A., Weiskopf D., Turtle L. (2020). Two Is Better Than One: Evidence for T-Cell Cross-Protection Between Dengue and Zika and Implications on Vaccine Design. Front. Immunol..

[56] Elong Ngono A., Shresta S. (2019). Cross-Reactive T Cell Immunity to Dengue and Zika Viruses: New Insights Into Vaccine Development. Front. Immunol..

[57] Gourinat A.C., O’Connor O., Calvez E., Goarant C., Dupont-Rouzeyrol M. (2015). Detection of Zika virus in urine. Emerg. Infect. Dis..

[58] Screaton G., Mongkolsapaya J., Yacoub S., Roberts C. (2015). New insights into the immunopathology and control of dengue virus infection. Nat. Rev. Immunol..

[59] Rivino L., Kumaran E.A., Thein T.L., Too C.T., Gan V.C., Hanson B.J., Wilder-Smith A., Bertoletti A., Gascoigne N.R., Lye D.C. (2015). Virus-specific T lymphocytes home to the skin during natural dengue infection. Sci. Transl. Med..

[60] Lee A., Park S.P., Park C.H., Kang B.H., Park S.H., Ha S.J., Jung K.C. (2015). IL-4 Induced Innate CD8+ T Cells Control Persistent Viral Infection. PLoS Pathog..

[61] Ozaki K., Spolski R., Feng C.G., Qi C.F., Cheng J., Sher A., Morse H.C., Liu C., Schwartzberg P.L., Leonard W.J. (2002). A critical role for IL-21 in regulating immunoglobulin production. Science.

[62] Konforte D., Simard N., Paige C.J. (2009). IL-21: An executor of B cell fate. J. Immunol..

[63] Rojas J.M., Avia M., Martín V., Sevilla N. (2017). IL-10: A Multifunctional Cytokine in Viral Infections. J. Immunol. Res..

[64] Pérez A.B., García G., Sierra B., Alvarez M., Vázquez S., Cabrera M.V., Rodríguez R., Rosario D., Martínez E., Denny T. (2004). IL-10 levels in Dengue patients: Some findings from the exceptional epidemiological conditions in Cuba. J. Med. Virol..

[65] Stewart C.A., Metheny H., Iida N., Smith L., Hanson M., Steinhagen F., Leighty R.M., Roers A., Karp C.L., Müller W. (2013). Interferon-dependent IL-10 production by Tregs limits tumor Th17 inflammation. J. Clin. Investig..

[66] Brandt E.B., Sivaprasad U. (2011). Th2 Cytokines and Atopic Dermatitis. J. Clin. Cell. Immunol..

[67] Geha R.S., Jabara H.H., Brodeur S.R. (2003). The regulation of immunoglobulin E class-switch recombination. Nat. Rev. Immunol..

[68] Zheng X., Yao J., Li B. (2016). Expression of TSLP and Downstream Molecules IL-4, IL-5, and IL-13 on the Eye Surface of Patients with Various Types of Allergic Conjunctivitis. J. Ophthalmol..

[69] Katial R.K., Bensch G.W., Busse W.W., Chipps B.E., Denson J.L., Gerber A.N., Jacobs J.S., Kraft M., Martin R.J., Nair P. (2017). Changing Paradigms in the Treatment of Severe Asthma: The Role of Biologic Therapies. J. Allergy Clin. Immunol. Pr..

[70] Hiraoka N., Yokote T., Nakayama-Ichiyama S., Takayama A., Iwaki K., Kobayashi K., Oka S., Miyoshi T., Akioka T., Takubo T. (2010). High fever and shock induced by interferon-γ and interleukin-6 produced by adult T-cell leukaemia/lymphoma cells. Leuk. Res..

[71] Fajgenbaum D.C., June C.H. (2020). Cytokine Storm. N. Engl. J. Med..

