# Supplementary material for: Detection of Anti-ZIKV NS1 IgA, IgM, and Combined IgA/IgM and Identification of IL-4 and IL-10 as Potential Biomarkers for Early ZIKV and DENV Infections in Hyperendemic Regions, Thailand
Source: Trop Med Infect Dis. 2023 May 17;8(5):284. doi: 10.3390/tropicalmed8050284 (PMC10222161; doi:10.3390/tropicalmed8050284)
Supplement: Supplementary file 1 [file tropicalmed-08-00284-s001.zip › tropicalmed-2292141-supplementary.pdf]

Tropical Medicine and Infectious Disease

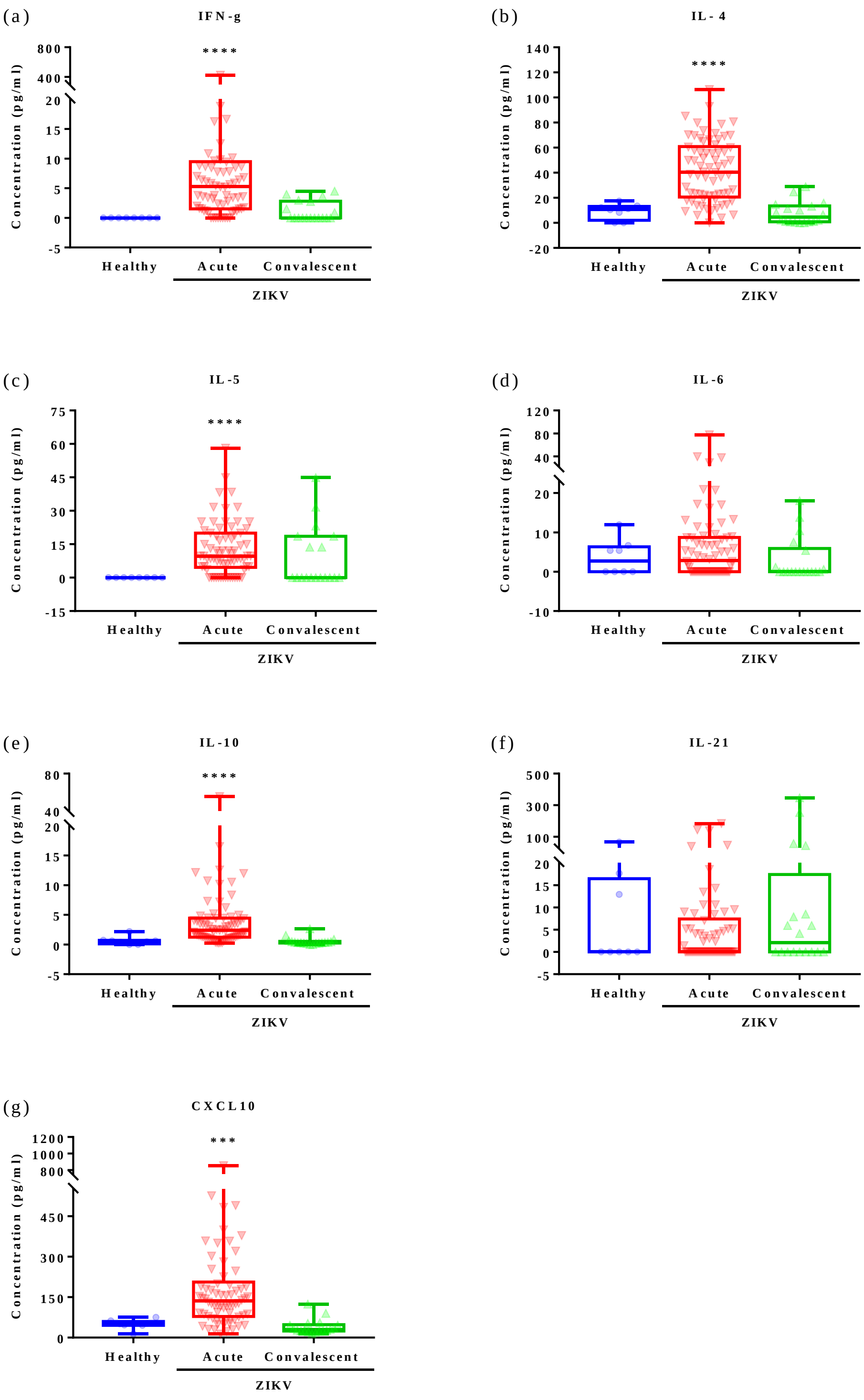

Figure S1. Levels of immune mediators in the acute and convalescent phases of ZIKV infection in comparison to healthy donors. All significant correlations are presented with the degree of significance indicated (* $p < 0.05$, ** $p < 0.01$, *** $p < 0.001$, **** $p < 0.0001$).

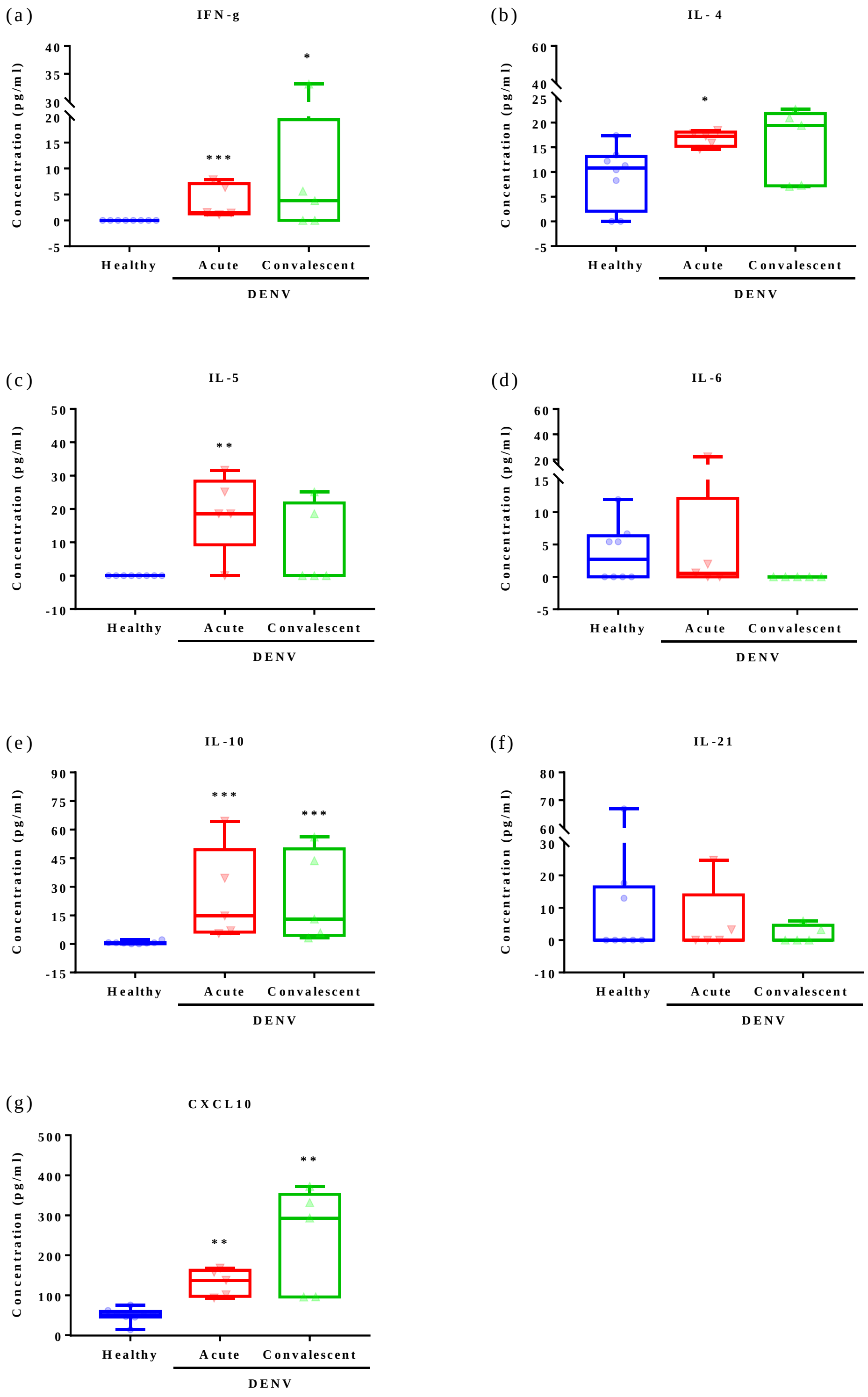

Figure S2. Levels of immune mediators in the acute and convalescent phase of DENV infection in comparison to healthy donors. All significant correlations are presented with the degree of significance indicated (* $p < 0.05$, ** $p < 0.01$, *** $p < 0.001$, **** $p < 0.0001$).

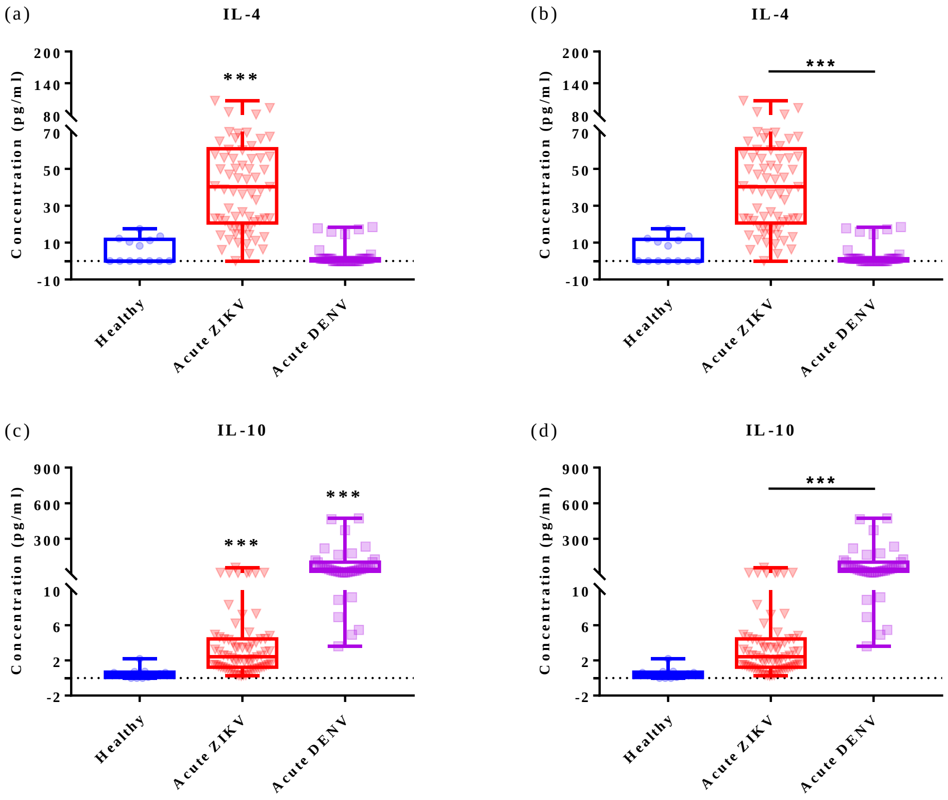

Figure S3. Levels of IL-4 and IL-10 in acute ZIKV cases (n = 70) and acute DENV cases (n = 40) in comparison to healthy donors (n = 13). (Note: * $p < 0.05$, ** $p < 0.01$, *** $p < 0.001$, **** $p < 0.0001$)

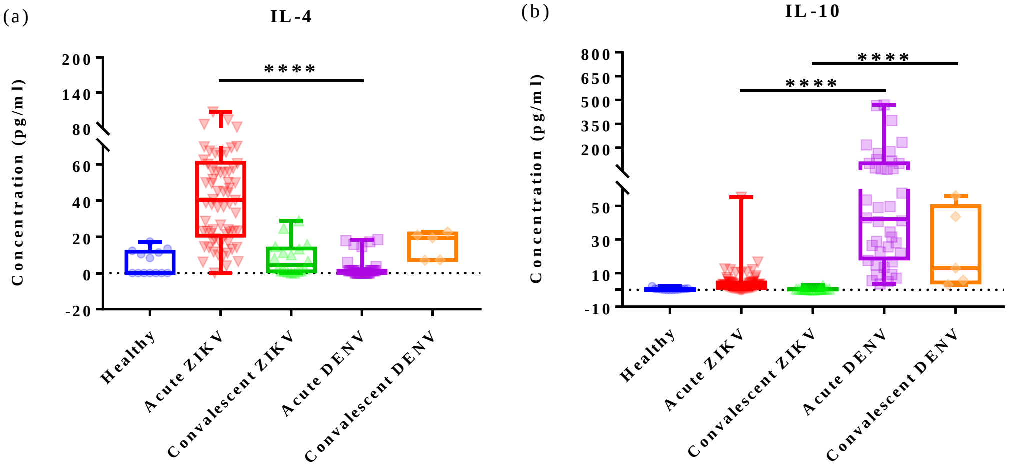

Figure S4. Differences in the levels of IL-4 and IL-10 in the acute and convalescent phases of ZIKV cases (acute, n = 70; convalescent, n = 18) compared with DENV cases (acute, n = 40; convalescent, n = 5). All significant correlations are presented with the degree of significance indicated (* $p < 0.05$, ** $p < 0.01$, *** $p < 0.001$, **** $p < 0.0001$).

Table S1. Changes in levels of IL-4 and IL-10 in the acute phase of infection among ZIKV-infected and DENV-infected cases compared to healthy donors.

Type of serum samples	Cytokine Concentration (pg/ml)	
	**IL-4**	**IL-10**
**Acute ZIKV**<br>(n = 70)	41.85 ± 24.85/2.97<br>40.44 (0-106.30)<br>35.93, 47.78	4.26 ± 7.06/0.84<br>2.44 (0.24-55.16)<br>2.58, 5.95
**Acute DENV**<br>(n = 40)	2.82 ± 5.49/0.87<br>1.29 (0-18.42)<br>1.06, 4.57	86.79 ± 116.20/18.37<br>42.02 (3.61-470.40)<br>49.63, 123.90
**Healthy donor**<br>(n = 13)	0 ± 6.63/1.84<br>0 (0-17.39)<br>1.62, 9.63	0.48 ± 0.58/0.16<br>0.40 (0-2.18)<br>0.13, 0.83

Note: Data are the values (in pg per ml) of mean ± SD (standard deviation)/SEM (standard error of the mean), median (minimum–maximum), and 95% CI (confidence interval), respectively. The results were analyzed by Mann–Whitney U test.

Table S2. Differences in the levels of IL-4 and IL-10 in the acute phase of infection among ZIKV-infected patients, DENV-infected patients, and healthy donors.

Type of serum samples	Type of Cytokine[a]	
	**IL-4**	**IL-10**
**Acute ZIKV**<br>vs<br>**Healthy donor**	< 0.001;<br>***	< 0.001;<br>***
**Acute DENV**<br>vs<br>**Healthy donor**	0.735;<br>*ns*	< 0.001;<br>***
**Acute ZIKV**<br>vs<br>**Acute DENV**	< 0.001;<br>***	< 0.001;<br>***

[a]All significant correlations are presented with the degree of significance indicated (* $p < 0.05$, ** $p < 0.01$, *** $p < 0.001$, **** $p < 0.0001$, *ns* = not significant); the results are analyzed by Mann-Whitney U test.

Tropical Medicine and Infectious Disease

MDPI

Table S3. Changes in levels of IL-4 and IL-10 in the acute and convalescent phases of infection among ZIKV-infected and DENV-infected cases compared to healthy donors.

Type of serum samples	Cytokine Concentration (pg/ml)	
	**IL-4**	**IL-10**
**Acute ZIKV**<br>(n = 70)	41.85 ± 24.85/2.97<br>40.44 (0-106.30)<br>35.93, 47.78	4.26 ± 7.06/0.84<br>2.44 (0.24-55.16)<br>2.58, 5.95
**Convalescent ZIKV**<br>(n = 18)	7.90 ± 8.77/2.07<br>4.41 (0-28.81)<br>3.54, 12.26	0.54 ± 0.63/0.15<br>0.35 (0-2.64)<br>0.23, 0.85
**Acute DENV**<br>(n = 40)	2.82 ± 5.49/0.87<br>1.29 (0-18.42)<br>1.06, 4.57	86.79 ± 116.20/18.37<br>42.02 (3.61-470.40)<br>49.63, 123.90
**Convalescent DENV**<br>(n = 5)	15.50 ± 7.66/3.43<br>19.43 (7.08-22.72)<br>5.99, 25.02	24.35 ± 23.99/10.73<br>13.08 (3.13-56.09)<br>-5.43, 54.14
**Healthy donor**<br>(n = 13)	0 ± 6.63/1.84<br>0 (0-17.39)<br>1.62, 9.63	0.48 ± 0.58/0.16<br>0.40 (0-2.18)<br>0.13, 0.83

Note: Data are the values (in pg per ml) of mean ± SD (standard deviation)/SEM (standard error of the mean), median (minimum–maximum), and 95% CI (confidence interval), respectively. The results were analyzed by Mann–Whitney U test.

Table S4. Differences in the levels of IL-4 and IL-10 in the acute and convalescent phases of infection among ZIKV-infected patients and DENV-infected patients.

Type of serum samples	Type of Cytokine[a]	
	**IL-4**	**IL-10**
**Acute ZIKV** vs<br>**Acute DENV**	< 0.0001;<br>****	< 0.0001;<br>****
**Convalescent ZIKV** vs<br>**Convalescent DENV**	0.0909;<br>*ns*	< 0.0001;<br>****

[a]All significant correlations are presented with the degree of significance indicated (* $p < 0.05$, ** $p < 0.01$, *** $p < 0.001$, **** $p < 0.0001$, *ns* = not significant); the results were analyzed by Mann–Whitney U test.
